# The lipidome of *Crithidia fasiculata*and its plasticity

**DOI:** 10.3389/fcimb.2022.945750

**Published:** 2022-10-28

**Authors:** Michela Cerone, Matthew Roberts, Terry K. Smith

**Affiliations:** Biomedical Science Research Complex (BSRC) Schools of Biology and Chemistry, University of St. Andrews North Haugh, St. Andrews, United Kingdom

**Keywords:** kinetoplastid, Crithidia, lipids, fatty acids, plasticity, oils, sugars

## Abstract

*Crithidia fasiculata* belongs to the trypanosomatidae order of protozoan parasites, bearing close relation to other kinetoplastid parasites such as *Trypanosoma brucei* and *Leishmania spp.* As an early diverging lineage of eukaryotes, the study of kinetoplastid parasites has provided unique insights into alternative mechanisms to traditional eukaryotic metabolic pathways. *Crithidia* are a monogenetic parasite for mosquito species and have two distinct lifecycle stages both taking place in the mosquito gut. These consist of a motile choanomastigote form and an immotile amastigote form morphologically similar to amastigotes in *Leishmania*. Owing to their close relation to *Leishmania, Crithidia* are a growing research tool, with continuing interest in its use as a model organism for kinetoplastid research with the added benefit that they are non-pathogenic to humans and can be grown with no special equipment or requirements for biological containment. Although comparatively little research has taken place on *Crithidia*, similarities to other kinetoplast species has been shown in terms of energy metabolism and genetics. *Crithidia* also show similarities to kinetoplastids in their production of the monosaccharide D-arabinopyranose similar to Leishmania, which is incorporated into a lipoarabinogalactan a major cell surface GPI-anchored molecule. Additionally, *Crithidia* have been used as a eukaryotic expression system to express proteins from other kinetoplastids and potentially other eukaryotes including human proteins allowing various co- and post-translational protein modifications to the recombinant proteins. Despite the obvious usefulness and potential of this organism very little is known about its lipid metabolism. Here we describe a detailed lipidomic analyses and demonstrate the possible placidity of *Crithidia’s* lipid metabolis. This could have important implications for biotechnology approaches and how other kinetoplastids interact with, and scavenge nutrients from their hosts.

## Introduction

*Crithidia fasciculata* is a non-human-infective kinetoplastid parasite phylogenetically related to the pathogenic forms *Trypanosoma brucei*, *Trypanosoma cruzi* and *Leishmania* spp (Kaufer et al., 2017). These parasites are the causative agents of Sleeping Sickness, Chagas Disease and Leishmaniasis. Collectively, with other Neglected Tropical Diseases (NTDs), they affect 1.7 billion people, from Africa to America to Asia. Lately, due to the climate change and global warming, cases of NTDs have been also registered in Southern Europe (Tidman et al., 2021; Elphick-Pooley and Engels, 2022). Despite the achievements recently made in the search for innovative drugs, and the successful elimination of at least one NTDs in 30 countries, a lot of work is still on the way to reach the goal of eradication of NTDs by 2030 (Elphick-Pooley and Engels, 2022). On this scenario, *C. fasciculata* has been used for decades as a model organism to study the biochemical, cellular, and genetic mechanisms that are unique to the members of the Trypanosomatidae family (Kipandula et al., 2018). This approach has created, and will, a concrete opportunity to try and identify, characterise, and validate novel drug targets (Kipandula et al., 2018).

There are numerous advantages of utilizing *C. fasciculata* as a model organism for the study of kinetoplastids’ metabolism. Firstly, they can be cultured in standard laboratory environment without requiring either BSL2 or BSL3 biosafety precautions (Tetaud et al., 2002; Kipandula et al., 2018). Secondly, these parasites grow rapidly at high densities in inexpensive undefined or fully defined serum-free media and at ambient temperature (Tetaud et al., 2002). Moreover, this single-cell system is easy to manipulate chemically and genetically, which render it amenable to biochemical and molecular analysis of complex pathways unique to infective and non- kinetoplastid parasites (Tetaud et al., 2002). Moreover, *C. fasciculata* are highly adaptable to very harsh and unusual conditions, and able to uptake and use the most various carbon sources as building blocks for their metabolic functions. This allows for the study of key and unique cellular pathways, which can be easily tuned and altered, at very low-cost. An exquisite example of a tuneable and plastic metabolic pathway is given by the biosynthesis of lipids and fatty acids (Parreira de Aquino et al., 2021). In fact, *C. fasciculta* as well as *T. brucei, T. cruzi and Leishmania*, possess a very vast and unique repertoire of enzymes, that play key roles in the synthesis of the most diverse lipids and fatty acids (Smith and Bütikofer, 2010; Booth and Smith, 2020; Parreira de Aquino et al., 2021). Despite the obvious usefulness and potential of this organism very little is known about its lipid metabolism.

Here we present a detailed analysis of the lipidome of *C. fasciculata* utilising a combination of genomic information and lipidomic mass spectrometry data to elucidate what they should be able to *de novo* synthesise and what they actually possess in their lipid membranes, this is followed up with a demonstration of and how this can be remodelled by the supplementation of the culture media with very cheap fat and/or carbohydrate sources.

## Results

### Phospholipid composition of *Crithidia fasiculata*

The study of lipid content and lipid metabolism of *Crithidia* has yet to be investigated, but the species are known to be composed of lipids common to eukaryotic species such as phospholipids, triglycerides, sterols and fatty acids (Meyer and Holz, 1966).

**Figure 1:**
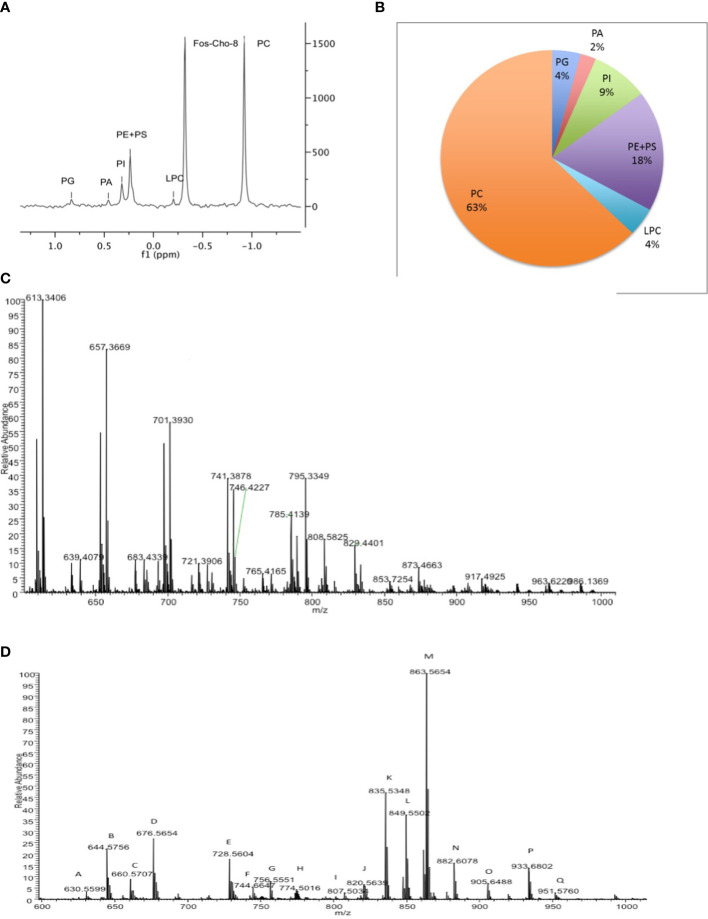
*Crithidia fasiculata* Phospholipid Composition **(A)** (Mwenechanya et al., 2017)P-NMR analysis of cellular lipid extract. **(B)** Quantitation of phospholipid content by head-group (Mwenechanya et al., 2017)P NMR. High resolution mass spectrometry survey scan of lipid extract in **(C)** positive mode and **(D)** negative mode.

To assess the distribution of phospholipids within *Crithidia*, lipid extracts were analysed by (Mwenechanya et al., 2017)P-NMR analysis (Sotirhos et al., 1986; Murgia et al., 2003; MacKenzie et al., 2009), with the inclusion of the *lyso*-choline analogue Fos-Cho-8 as an internal standard. Lipid classes were assigned based upon chemical shift and integration of lipid standards in relation to Fos-Cho-8 (**Figures 1A**, **S1**). From (Mwenechanya et al., 2017)P-NMR analysis of *Crithidia* total cell lipid extracts (**Figure 1A**), *Crithidia* are shown to be capable of producing all of the main phospholipid species expected of a eukaryote. Of these, phosphatidylcholine (PC) is the most abundant representing ~63% of the total, while the combination of phosphatidylethanolamine (PE) and phosphatidylserrine (PS) represents ~18% of the total, with the former being the majority based upon subsequent mass spectrometric analyses (**Figure 1B**). High resolution mass spectrometry survey scan in both positive (**Figure 1C**) and negative (**Figure 1D**) ion mode were done allowing an initial identification of the phospholipid species based upon the accurate mass (**Tables S1**, **S2** respectively). Unusually for a eukaryote, a number of PE lipds were identified as having odd chain fatty acid content. Additionally, as unlike mammalian cells the sphingolipid inositolphosphorylceramide (IPC) is formed instead of sphingomyelin (SM), both of these will be investigated later.

### Inositol lipid metabolism

The production of phosphatidylinositol (PI) lipids in kinetoplastids is reliant upon two routes of sourcing inositol: *de-novo* synthesis from glucose or uptake of *myo*-inositol from an extracellular source through a transporter (Martin and Smith, 2006a; Martin and Smith, 2006b). PI lipids have a number of downstream roles such as undergoing phosphorylation to form phosphorylated phosphatidylinositol (PIPn) or undergoing head-group addition (inositol-phosphate) to a ceramide, forming the sphingolipid IPC. Additionally, PI species are also incorporated into glycolipids such as glycosylinositolphospholipids (GIPLs) and GPI-anchored proteins (Ferguson, 1997). *Crithidia* are known to produce the monosaccharide D-arabinopyranose, which it is utilized in the production of a lipoarabinogalactan (LAG) similar to the lipophosphoglycans (LPG) of *Leishamania (* Schneider et al., 1996), both of which are present at high abundance as cell surface GPI-anchored molecule (Ferguson, 1997). The LAG present in *Crithidia* differs from LPG in the composition of the GPI anchor, incorporating an IPC lipid moiety as opposed to a PI (Schneider et al., 1996).

#### Genomic analysis of Inositol lipid metabolism

**Figure 2:**
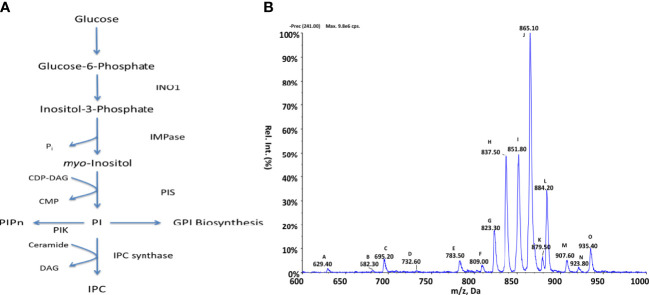
Inositol metabolism in Crithidia **(A)** An overview of inositol metabolism in kinetoplasts. (Key – INO1, Inositol-3-phosphate synthase; IMPase PIK, Phosphatidylinositol kinase; PIS, Phosphatidylinositol synthase; IPC synthase, Inositol phosphorylceramide synthase; PIPn, Phosphatidylinositol phosphate; CDP-DAG, cytidine diphosphate-diacylglycerol; CMP, cytidine monophosphate; IPC, phosphorylceramide.) **(B)** ESI-MS-MS negative parent ion scan of PI lipids from *C. fasiculata* lipid extract, scanning for m/z 241. Peaks are plotted as their relative intensity (%) to that of the largest peak in the spectrum and in terms of their mass to charge (m/z) ratio. (Species identities are given in **Supplementary Table 4**).

To examine the PI biosynthetic pathways present within *Crithidia*, the *TriTrypDB* database (Aslett et al., 2010) was utilised to examine the genome of *Crithidia* for homologues of enzymes known to be involved in inositol metabolism from other kinetoplastid species (**Figure 2A**; **Table S3**). The genes identified show that *Crithidia* possess putative genes for the synthesis of inositol-3-phosphate from glucose (inositol-3-phosphate synthase, INO1) and two putative genes for an inositol monophosphatase (IMPase), as it is for the other kinetoplastids, suggesting the ability to *de-novo* synthesise *myo*-inositol from glucose (Martin and Smith, 2006a). A single putative gene for phosphatidylinositol synthase was also identified (Martin and Smith, 2006b), along with several PI and PIP kinases, suggesting the production and downstream use of PIP species as potential secondary signalling molecules, which seems to be ubiquitous in eukaryotes. Interestingly, a close homologue inositol phosphorylceramide synthase such as those in *Leishmania* spp. and *T. cruzi* was not found within the genome (Zhang, 2003). Instead, a single putative gene was found with homology to the series of sphingolipid synthases (SLS) present within other kintetoplasts. The members of the TbSLS synthase family has been characterised as having >90% identity (Goren et al., 2011), with substrate selectivity determined by the characteristics of three residues within the active site. Therefore, the conservation of the CfSLS active site with that of TbSLS1 confirms its function as an IPC synthase.

Inositol lipid composition data *Crithidia* lipid extracts were examined *via* ESI-MS-MS. Parent ion scanning for the inositol 1,2-cyclic-phosphate head (m/z 241) in the negative ion mode, was utilised to identify all PI species present (**Figure 2B**). PI species highlighted by parent ion scanning were assigned based upon accurate mass. Identification of the acyl-content of species was achieved by collision-induced fragmentation of species in the negative ion mode, producing daughter fragment spectra (**Table S4**).

By parent ion scanning for inositol 1,2-cyclic-phosphate at m/z 241 (**Figure 2A**), numerous peaks can be observed with peak J (m/z 865) representing PI 36:*n* (**Table S4**), which constitute the most abundant lipid species.

The lipidomic analysis above shows that numerous PI molecular species are produced by *Crithidia*, in addition several *lyso*-PI, PIP and IPC species. The presence of the IPC 38:0 (m/z 837), in conjunction with the other main IPC series: IPC 34:*n* (m/z 780) and IPC 36:*n* (m/z 808), confirm that *Crithidia* do possess an active IPC synthase. IPC was found to be the only sphingolipid identified from *Crithidia*, with no SM or EPC observed. From the daughter ion scanning of identified IPC species, the acyl content from IPC could not be reliably determined due to a low signal of characteristic fragments suggesting acyl loss. Likewise, attempts to investigate the long chain base composition in the positive ion mode did not prove conclusive. However, for the IPC species 36:0 a weak ion is observed in the positive mode fragmentation spectra at m/z 284, representing a d18:0–H_2_O. In *L. major* the main IPC species produced have been shown to contain C16 as the long chain base, inferring that *L. major* SPT exhibits a preference for myristoyl-CoA as a substrate over the palmitoyl-CoA utilised in other kinetoplasts (Zhang, 2003; Hsu et al., 2007).

Other major species observed are peaks: H (m/z 837), I (m/z 851), J (m/z 865) and L (m/z 884) representing the series IPC 38:*n*, PI a-36:*n*, and PI 38:*n* respectively.

Similarly, the presence of PIP species would suggest that the PI kinases as identified in the genomic analysis above are indeed functional. As the observed PI and PIP species were found to be composed of a mixture of diacyl and acyl-alkyl moieties, it is likely that PI/PIP species are undergoing acyl remodelling and may possess numerous acyl-transferases.

#### Choline lipid metabolism

**Figure 3:**
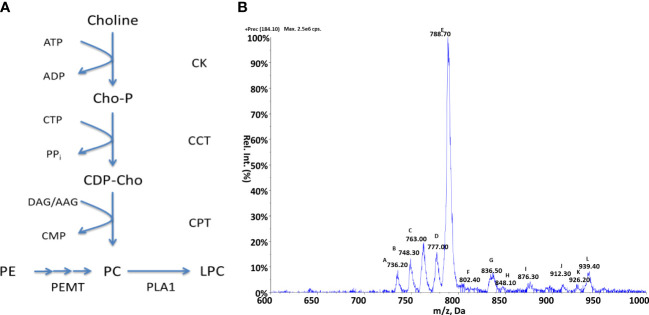
Choline metabolism in *Crithidia* **(A)** An overview of PC metabolism in eukaryotes. Key - CCT, Choline-phosphate cytidylyltransferase; CK, Choline kinase; CPT, Choline phosphotransferase; PEMT, Phosphatidylethanolamine N-methyltransferase; PLA1, Phospholipase A1; AAG, alkyl-acylglycerol; ADP, adenosinediphosphate; ATP, adenosine-triphosphate; CDP-Cho, cytidine diphosphate-choline; CMP, cytidine monophosphate; CTP, cytidine-triphosphate; DAG, diacylglycerol; Cho-P, choline phosphate; LPC, lyso-phosphatidylcholine. **(B)** ESI-MS-MS positive parent ion scan of PC lipids from *C. fasiculata* lipid extract, scanning for m/z 184. Peaks are plotted as their relative intensity (%) to that of the largest peak in the spectrum and in terms of their mass to charge (m/z) ratio. Species identities are given in **Table S6**.

The production of PC lipids in kinetoplastids can occur *via* two possible pathways (Gibellini and Smith, 2010): *de-novo* synthetic routes, either from choline *via* the Kennedy pathway or from the sequential methylation of PE species *via* the activity of a S-adenosyl-L-methionine (SAM) dependent methyltransferases, these pathways are summarised in **Figure 3A**. Protozoan parasites such as *Leishmania* and *Plasmodium (* Lehane et al., 2004) have been characterised as possessing choline transporters from the extracellular environment. Differing from these species, *T. brucei* has been shown to be auxotrophic for choline (Rifkin et al., 1995) relying upon the uptake exogenous *lyso*-PC. As with PE metabolism, the Kennedy pathway and methylation of PE are yet to be examined in *Crithidia* and this section aims to investigate which of these metabolic routes are present in *Crithidia* and which forms the main route of PC biosynthesis.

#### Genomic analysis of choline lipid metabolism

To examine the PC biosynthetic pathways present within *Crithidia*, the *TriTypDB* database (Aslett et al., 2010) was utilised to examine the genome of *Crithidia* for homologues of enzymes known to be involved in PC metabolism from other kinetoplast species.

The genes identified (**Table S5**) show that *Crithidia* possess putative genes for choline kinase, choline phosphotransferase, and choline-phosphate cytidyltransferase, which make up the Kennedy pathway. Two putative methyltransferases were also identified in *Crithidia* and are located in different locations within the genome. As these genes do not show any sequence homology to each other, it is likely that these enzymes show different specificities to each other. Based upon homology with PEMT enzymes characterised from *L. major*, it is likely that CfaC1_34_5650 represents a class 2 PEMT which catalyses the initial methylation of PE, with CfaC1_34_4100 a class 1 PEMT responsible for the addition of a 2^nd^ and 3^rd^ methyl group (Bibis et al., 2014).

#### Choline lipid composition data

*Crithidia* lipid extracts were produced using the modified Bligh-Dyer extraction from steady state cell cultures and examined *via* ESI-MS-MS. Parent ion scanning for the choline phosphate head group (m/z 184) in the positive ion mode, was utilised to identify all PC species present (**Figure 3B**). The PC species highlighted by parent ion scanning were assigned based upon accurate mass data. Identification of the acyl-content of species was achieved by collision-induced fragmentation of species in the negative ion mode, producing daughter fragment spectra. (**Table S6**)

The parent ion data (**Figure 3B**) shows a number of PC species, with the most abundant being peak E (m/z 788) which represents the series PC 36:*n*. Other major species were the peaks: A (m/z 736), B (m/z 748), C (m/z 763) and D (m/z 777) representing the PC series: 32:*n*, a-34:*n*, 34:*n* and a a-36:*n* respectively. The minor species represented by the peaks J, K and L are thought to be sodium adducts of minor species. As previously observed with PI species, the PC species identified were found to be composed of a mixture of diacyl and acyl-alkyl moieties.

In keeping with the aforementioned similarity of *Crithidia* to *Leishmania* spp. and *T. cruzi*, no sphingomyelin species were observed further suggesting that IPC is the sole sphingolipid produced. By comparison of the observed PC species to PE species (as discussed later), the activity of the PEMTs identified from genetic analyses above can be confirmed. This is evidenced by the correlation between the major PE series observed as: 34:*n*, a-36:*n* and 36:*n* (**Figure 3B**), with the corresponding major PC series consisting: 34:*n*, a-36:*n* and 36:*n* also. Therefore, it is likely that the methylation of PE species is the major route for PC synthesis as in *L. major (* Bibis et al., 2014).

### Ethanolamine lipid metabolism

**Figure 4:**
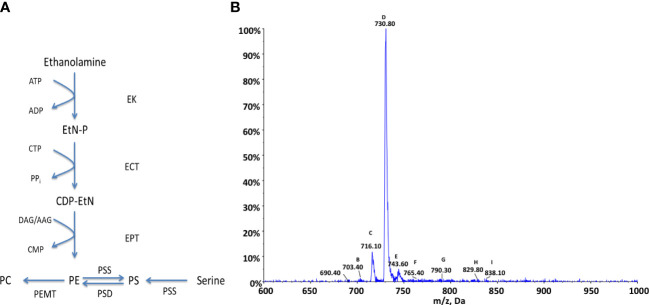
Ethanolamine metabolism in *Crithidia* **(A)** An overview of PE metabolism in most eukaryotes. Key - ECT, Ethanolamine-phosphate cytidylyltransferase; EK, ethanolamine kinase; EPT, Ethanolamine phosphotransferase; PEMT, Phosphatidylethanolamine N-methyltransferase; PSD, Phosphatidylserine decarboxylase; PSS, Phosphtidylserine synthase; AAG, alkyl-acylglycerol; ADP, adenosinediphosphate; ATP, adenosine-triphosphate; CDP-EtN, cytidine diphosphate-ethanolamine; CMP, cytidine monophosphate; CTP, cytidine-triphosphate; DAG, diacylglycerol; EtN-P, ethanolamine phosphate. **(B)** ESI-MS-MS negative parent ion scan of PE lipids from *C. fasiculata* lipid extract, scanning for m/z 196. Peaks are plotted as their relative intensity (%) to that of the largest peak in the spectrum and in terms of their mass to charge (m/z) ratio. (Species identification in **Table S8**).

As with choline the production of PE lipids in kinetoplastids can occur *via* two main pathways: either *de-novo* synthesis from ethanolamine *via* the Kennedy pathway or the decarboxylation of PS species *via* serine decarboxylase. PE species can also be utilised in the production of PC species *via* the subsequent activity of a SAM dependant methyltransferase. PE can also undergo head-group exchange with serine to produce PS species, these pathways are summarised in **Figure 4A**. In *T. brucei* it has been shown that the Kennedy pathway is essential and the sole synthetic route to PE, despite the presence of a serine decarboxylase, which contributes very little to PE synthesis (Gibellini et al., 2009). An alternate route, which has been shown to be highly active in *Leishmania* is the breakdown of phosphorylated sphingoid bases by sphingosine-1-phosphate lyase to produce its main source of ethanolamine phosphate (Pawlowic et al., 2016), as they are unable to take up ethanolamine from an extracellular source.

#### Genomic analysis of ethanolamine lipid metabolism

To examine the PE biosynthetic pathways present within *Crithidia*, the TriTrypDB database (Aslett et al., 2010) was utilised to examine the genome of *Crithidia* for homologues of enzymes known to be involved in PE metabolism from other kinetoplast species (**Table S7**).

The genes identified show that *Crithidia* possess putative genes for ethanolamine kinase, ethanolamine phosphotransferase, and ethanolamine-phosphate cytidyltransferase, which make up the Kennedy pathway. While, *T. brucei* lack a methyl-transferase to convert between PC and PE species, two putative methyltransferases were identified in *Crithidia* showing similarity to those produced in various *Leishmania* species as previously described. It is unclear from the database if the putative PS synthase acts similar to a type one or two synthase and would need to be determined experimentally although from the amino acid sequence it does show more similarity to a type-II head-group exchange PSS. Also identified was a putative phosphatidylserine decarboxylase suggesting *Crithidia* are additionally capable of producing PE from the decarboxylation of PS species.

#### Ethanolamine lipid composition data

*Crithidia* lipid extracts were produced using the modified Bligh-Dyer extraction from steady state cell cultures and examined *via* ESI-MS-MS. Parent ion scanning for the dehydro-ethanolaminephosphoglycerol (m/z 196) in the negative ion mode, was utilised to identify all PE species present (**Figure 4B**). The PE species highlighted by parent ion scanning were assigned based upon accurate mass data. Identification of the acyl-content of species was achieved by collision-induced fragmentation of species in the negative ion mode, producing daughter fragment spectra. Despite previous data showing PE to be one of the most abundant lipid classes in *Crithidia*, this analysis would suggest that there are only three main PE species that are abundantly produced by the organism. This could in part be due to ion suppression within the mass spectrometer leading to a reduced sensitivity for the minor species. The most abundant of which is represented by peak D (m/z 730), corresponding to the series PE a-36:n (**Table S8**). The other major species are peaks C (m/z 716) and E (m/z 743), which correspond to the series PE 34:n, and PE 36:n respectively.

As has been previously discussed, it would appear likely that PE represents a major route to PC by the activity of two PEMT enzymes. This is reflected in the main series of PC which correspond with those of PE, namely the 34:*n* a-36:*n* and 36:*n* series. Ethanolamine species identified were found to be composed of a mixture of diacyl and acyl-alkyl moieties.

*Crithidia* extracts were found to contain an unusually high proportion of odd carbon containing fatty acids, including the major PE species (m/z 730), assigned as PE 35:0. The acyl composition of the observed species was confirmed by ESI-MS-MS daughter fragmentation spectra, (**Figure S2**), including the large peak at m/z 295 represents a C19Δ fatty acid and ions characteristic of the molecular species with a loss of C19Δ can be observed in both the acyl (m/z 434) and ketene forms (m/z 450).

The production of cyclopropyl C19Δ fatty acid is predominantly observed in bacterial membranes (Grogan and Cronan, 1997), but has also been observed in certain species of *Leishmania* and is thought to act as a virulence factor (Oyola et al., 2012).

To investigate the distribution of the C19Δ fatty acid within *Crithidia* lipids, parent ion scanning (m/z 295) over the mass range m/z 700-800, was performed to identify C19Δ containing species (**Figure S3**). The composition of the lipid species identified was subsequently assigned by high-resolution ESI-MS accurate mass measurements (**Table S8**).

The other main series identified was PE a-35:*n* (m/z 714), alongside the minor species PE a-37:*n* (m/z 742) and PE 37:n (m/z 756). The species shown at m/z 751 was revealed to be the [M + Cl]^−^ adduct of PE a-16:0/C19Δ *via* fragmentation. Likewise the peak at m/z 764.80 is representative of the [M + Cl]^−^ adduct of PE 16:0/C19Δ.

The incorporation of C19Δ into PE species is in line with the observations by Hildebrand and Law (1964) where C19Δ were primarily observed in PE species from bacteria. PC species containing C19Δ were reported in *Agrobacterium tumefaciens (* Hildebrand and Law, 1964), however no PC species containing cyclopropyl C19Δ fatty acid were observed in *Crithidia* lipids. Given the large proportion (~10%) of C19Δ fatty acid within total cell extracts, it is unexpected that relatively few C19Δ lipid species are observed. As *Crithidia* are non-pathogenic to humans, the high abundance of C19Δ lipids are likely to possess a structural role as opposed to acting as a virulence factor. Equally surprising is the inclusion of C19Δ into alkyl-acyl species, as only diacyl lipids have been previously reported from bacteria (Hildebrand and Law, 1964; Grogan and Cronan, 1997). This would suggest that the cyclopropyl fatty acid synthase (CFAS) is only selective for the acyl chain at the sn_2_- position, which is in keeping with the previously reported selectivity from bacteria (Grogan and Cronan, 1997).

We and others have shown that *L. infantum* lipids, also have C19Δ containing PEs (Vincent et al., 2014; Mwenechanya et al., 2017), specifically series: a-35:*n*, a-37:*n*, 39:*n* in addition to 35:1 and a-41:6. These findings are in accordance with the C19Δ-PE species reported here, highlighting the potential similarity between the *Leishmania* and *Crithidia* CFAS. In a recent report by Hsu et al. ( Hsu et al., 2014), novel C19Δ-PE species were characterisation from *L. infantum* as PE a-35:3 (m/z 712), PE a-35:2 (m/z 714) and PE a-36:2 (m/z 728). While species were observed at similar masses to those described here, the assigned acyl composition is not in agreement of the above.

#### Serine lipid metabolism

**Figure 5:**
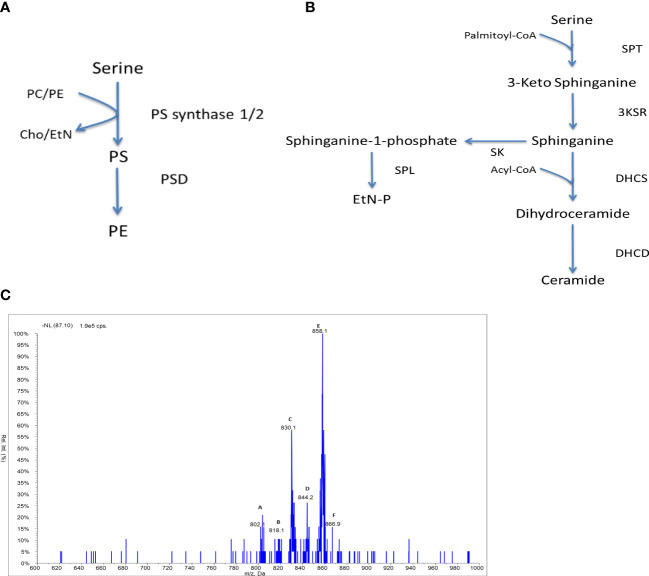
Serine lipid metabolism in *Crithidia:* An overview of **(A)** PS synthesis in eukaryotes, **(B)** ceramide synthesis in kinetoplastids. Key - 3KSR, 3-Ketosphinganine reductase; DHCD, Dihydroceramide desaturase; DHCS, Dihydroceramide synthase; PSD, Phosphatidylserine decarboxylase; SK, Sphingosine kinase; SPL, Sphingosine-1-phosphate lyase; SPT, Serine palmitoyltransferase; Cho, Choline; EtN, Ethanolamine; EtN-P, ethanolamine phosphate; PC, phosphatidylcholine; PE, phosphatidylethanolamine; PS, phosphatidylserine. **(C)** ESI-MS-MS neutral loss scan of PS lipids from *C. fasiculata* lipid extract, scanning for m/z 87 in the negative mode. Peaks are plotted as their relative intensity (%) to that of the largest peak in the spectrum and in terms of their mass to charge (m/z) ratio. (Species identification in **Table S10**).

For the production of PS, eukaryotes have been shown to utilise two serine exchange enzymes (**Figure 5A**), showing specificities for PC (PS-synthase 1, PSS1) and PE (PS-synthase 2, PSS2). In kinetoplastids as a whole, very little is known about the production of PS. The *T. brucei* PSS has recently been characterised as a type-II PS synthase (Farine et al., 2017), which shows a high level of homology to those putatively identified in both *Leishmania* and *T. cruzi*. It is unclear whether the mechanism of PS synthesis occurs in *Crithidia via* head exchange as above or instead proceeds similar to that of the bacterial PS-synthase.

Another main area of serine metabolism is the *de novo* synthesis of ceramides (**Figure 5B**), beginning with the condensation of serine and palmitoyl-coenzyme A (palmitoyl-CoA) by a serine palmitoyltransferase (SPT) forming 3-keto-sphinanine, which is subsequently reduced into the sphingoid base sphinganine. The *de-novo* synthesis of ceramide has been shown to be active in *T. brucei (* Sutterwala et al., 2007) where inhibition of SPT was identified as essential to the parasite. In *Leishmania*, the activity of SPT has been shown to be essential for viability due to a dependence upon the catabolism of sphingoid bases as a primary source of ethanolamine (Sutterwala et al., 2007; Zhang et al., 2007; Pawlowic et al., 2016).

#### Genomic analysis of serine lipid metabolism

To examine the serine lipid metabolic pathways present within *Crithidia*, the TriTryp database (Aslett et al., 2010) was utilised to examine the genome of *Crithidia* for homologues of enzymes known to be involved in serine lipid metabolism from other kinetoplastid species (**Table S9**). The genes identified show that *Crithidia* possess a PS synthase showing a high degree of homology to the recently characterised TbPSS2, similar to those of *Leishmania* spp and other kinetoplasts. This was further confirmed by a sequence alignment for the conserved CDP-DAG-phosphotransferase motif from bacterial PS-synthases, which found no sequence homology. A putative phosphatidylserine decarboxylase was also identified, suggesting *Crithidia* are potentially capable of producing PE from the decarboxylation of PS species.

From genomic analysis are putative enzymes for the *de novo* synthesis of ceramide. In *T. brucei*, there is a family of four sphingolipid synthases (SLS 1-4) which show specificity for the production of sphingomyelin (SM, SLS 3/4), ethanolamine phosphoceramide (EPC, SLS 2, SLS3/4) and inositol phosphoceramide (IPC, SLS 1) (Sutterwala et al., 2008; Mina et al., 2009; Smith and Bütikofer, 2010) alongside ceramide species. *Leishmania* however, only produce IPC sphingolipids, utilising an IPC-synthase similar in function to those characterised in fungi (Denny et al., 2006). Despite this, *L. major* still possess activity similar to a neutral sphingomyelinase (nSMase), derived from an IPC hydrolase referred to as an inositol phosphsphingolid phospholipase C-like protein (ISCL) due to similarities in its mechanism to phospholipase-C enzymes (Zhang et al., 2009). This protein is required for the degradation of IPC, but has been shown to have a greater activity for sphingomyelin (Zhang et al., 2009), suggesting a possible role in virulence. The *T. brucei* homologue is also able to cleave both SM and IPC and its localisation and role are different between the two main life cycle stages (Young and Smith, 2010; Dickie et al., 2019).

In *Crithidia* a single putative IPC-synthase was identified showing similarity to that in *Leishmania* and the SLS 1 in *T. brucei*. *Crithidia* were also found to possess a putative ISCL, suggesting an nSMase with dual specificity for SM/IPC as found in *Lesihmania*. Additionally, a putative SPL enzyme was found suggesting that *Crithidia* can use the catabolism of ceramides as a source of ethanolamine.

#### Serine lipid composition data

*Crithidia* lipid extracts contain relative low levels of PS. When examined *via* ESI-MS-MS, using neutral loss scanning for the dehydro-serine (m/z 87) in the negative ion mode, several PS species were identified (**Figure 5C**). The PS species highlighted by parent ion scanning were assigned based upon accurate mass data. Identification of the acyl-content of species was achieved by collision-induced fragmentation of species in the negative ion mode, producing daughter fragment spectra (**Table S10**).

Compared to other lipid classes, few PS species are observed from the neutral loss scanning (**Figure 3**.32), suggesting that PS represents a minor lipid class as seen in other kinetoplastid species. The major species are peak E (m/z 858) representing the series PS 42:*n* (table 3.13) and peak C (m/z 830) which represents the series PS a-40:*n*. The minor species observed are of the series: a-38:*n* (m/z 803), 38:*n* (m/z 818), 40:*n* (m/z 844) and 42:*n* (m/z 866).

The low abundance of PS species could suggest that PS production is highly regulated, similar to observations from *T. brucei* (Smith and Bütikofer, 2010; Williams et al., 2012). Alternately this could infer that there is a high turnover of PS species by the serine decarboxylase to PE as identified above. However, the acyl distribution of the PS species does not correlate well with the major PE species (predominantly PE a-36:*n*) suggesting that PS decarboxylation may not represent the main route towards the synthesis of PE species.

#### Fatty acid metabolism

**Figure 6:**
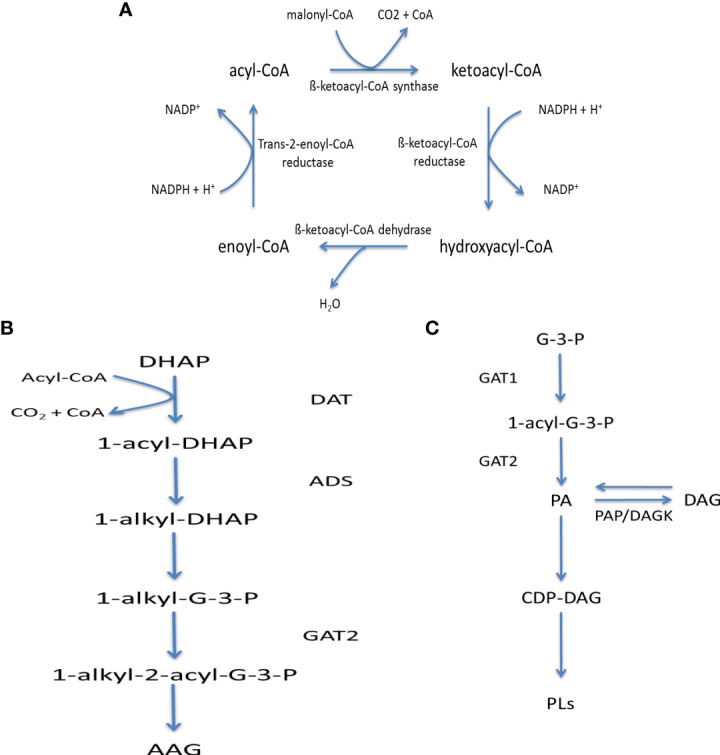
Fatty acid metabolism in kinetoplastids: **(A)** Fatty acid elongase mechanism, **(B)** Acyl-alkyl glycerol synthesis and **(C)** Phosphatidic acid synthesis and metabolism. Key – ADS, 1-Alkyl-dihydroxyacetonephosphate synthase; DAT, Dihydroxyacetonephosphate acyltransferase,; DAGK, DAG kinase; GAT1, Glycerol-3-phosphate acyltransferase; GAT2, 1-Acyl-sn-glycerol-3-phosphate acyltransferase; PAP, Phosphatidic acid phosphatase; AAG, Alkyl-acyl glycerol; CoA, Co-enzyme A; DAG, Diacylglycerol; G-3-P, Glycerol-3-phosphate; PA, Phosphatidic Acid; PLs, Phospholipids.

Kinetoplasts were originally thought to utilise a bacterial type II fatty acid synthase for the *de novo* synthesis of fatty acids. Instead, *T. brucei* was shown to produce the bulk of its fatty acids *via* a system of elongases (ELOs) to facilitate a requirement of myristate in the bloodstream form for use in fatty acid remodelling and myristate exchange processes of GPIanchors (Paul et al., 2001). Showing a close similarity to type I and type II FA-synthase pathways, the elongase mechanism consists of four ELOs which utilise CoA rather than ACP (**Figure 6A**). ELOs 1-3 are found in a tandem array and show specificity for certain chain lengths with ELO 1 extending C4 to C10, ELO2 extending C10 to C14 and ELO3 extending C14 to C18, ELO4 has been shown to extend C20:4 to C22:4 (Lee et al., 2006). Similar elongase mechanisms have been identified in *Leishmania* spp and *T. cruzi* (Tripodi et al., 2006; Livore et al., 2007), likewise showing specificities for differing acyl-lengths in addition to the production of poly-unsaturated fatty acids.

To allow for their incorporation into further metabolic processes, fatty acids undergo activation to acyl-CoA derivatives *via* acyl-CoA synthetases (ACS), which possess specificities for varying chain lengths. Once activated FAs can be involved in the biosynthesis of protein-lipidation, i.e. N-myristylation and S-palmitylation, as well as lipids by combination with either dihydroxyacetone-phosphate or glycerol-3-phosphate, produced from the catabolism of sugars, i.e. glycolysis, producing acyl-alkyl glycerol-phosphate or phosphatidic acid (**Figures 6B, C** respectively). The latter of which, can in turn be either dephosphorylated to DAG or activated into the high-energy donor CDP-DAG.

Fatty acid synthesis, activation and subsequent metabolism has yet to be examined in *Crithidia* and this section aims to investigate the question: which is the main route of fatty acid biosynthesis, *de novo* synthesis or uptake and potential elongation and/or desaturation in *Crithidia*?

#### Genomic analysis of fatty acid metabolism

To examine the fatty acid synthesis and activation pathways present within *Crithidia*, the TriTryp database (Aslett et al., 2010) was utilised to examine the genome of *Crithidia* for homologues of enzymes known from other kinetoplastid species.

The genes identified (**Table S11**) show that *Crithidia* possess several putative acyl-CoA synthases alongside a putative acyl-CoA carboxylase (ACC), suggesting the ability to produce the malonyl-CoA precursor required for *de-novo* FA synthesis, probably *via* an elongase mechanism. If this mechanism can be shown to be active it would be likely that these enzymes are also responsible for producing the butyl-CoA primer to initiate synthesis by the elongase system. Putative elongases were also identified showing similarities to the ELO 1-4 identified in *T. brucei*, however the extra elongases in *Crithidia* suggest additional specificities and/or cellular locations to both saturated and unsaturated fatty acids.

Similar to both *T. brucei* and *L. major* (Paul et al., 2001; Lee et al., 2007), genes were identified (**Table S11**) for a type II fatty acid synthase (FAS), including; β-ketoacyl-ACP reductase (KAR), Enoyl-ACP reductase (ENR), β-ketoacyl-ACP synthase (KAS) and β-ketoacyl-ACP dehydratase (DH). Based upon the presence of a putative acyl carrier protein (ACP), it is possible that C*rithidia* can form malonyl-ACP to be used in a type II FAS mechanism. Although a malonyl-CoA : ACP transacylase (MAT) was not identified, *Crithidia* possess an acyl-transferase like protein similar to that reported in *T. brucei* to possess MAT function (van Weelden et al., 2005). If active, this FAS type II system may act similarly to that observed in *T. brucei* which only forms a minor contribution to total fatty acid synthesis (Stephens et al., 2007), which is likely to be mitochondrial in location and may only serve for the *de novo* synthesis of lipoic acid.

*Crithidia* as demonstrated earlier and below, possess a cyclopropyl fatty acid synthase (CFAS), similar to that reported in *L. infantum*, where it was shown to act as a virulence factor (Oyola et al., 2012). It is unlikely that CFAS is involved in virulence within *Crithidia*, instead the role of cyclopropyl fatty acids is more likely to be involved in a structural role within membranes giving resistance to environmental stresses/changes similar to those observed in bacteria (Grogan and Cronan, 1997). The CFAS mechanism involves the transfer of a methyl group from a S-adenosyl-L-methionine (SAM) donor to a carbon-carbon double bond in a fatty acid chain namely C18:1 to form C19Δ.

#### Fatty acid profile of *Crithidia fasciculata*

**Figure 7:**
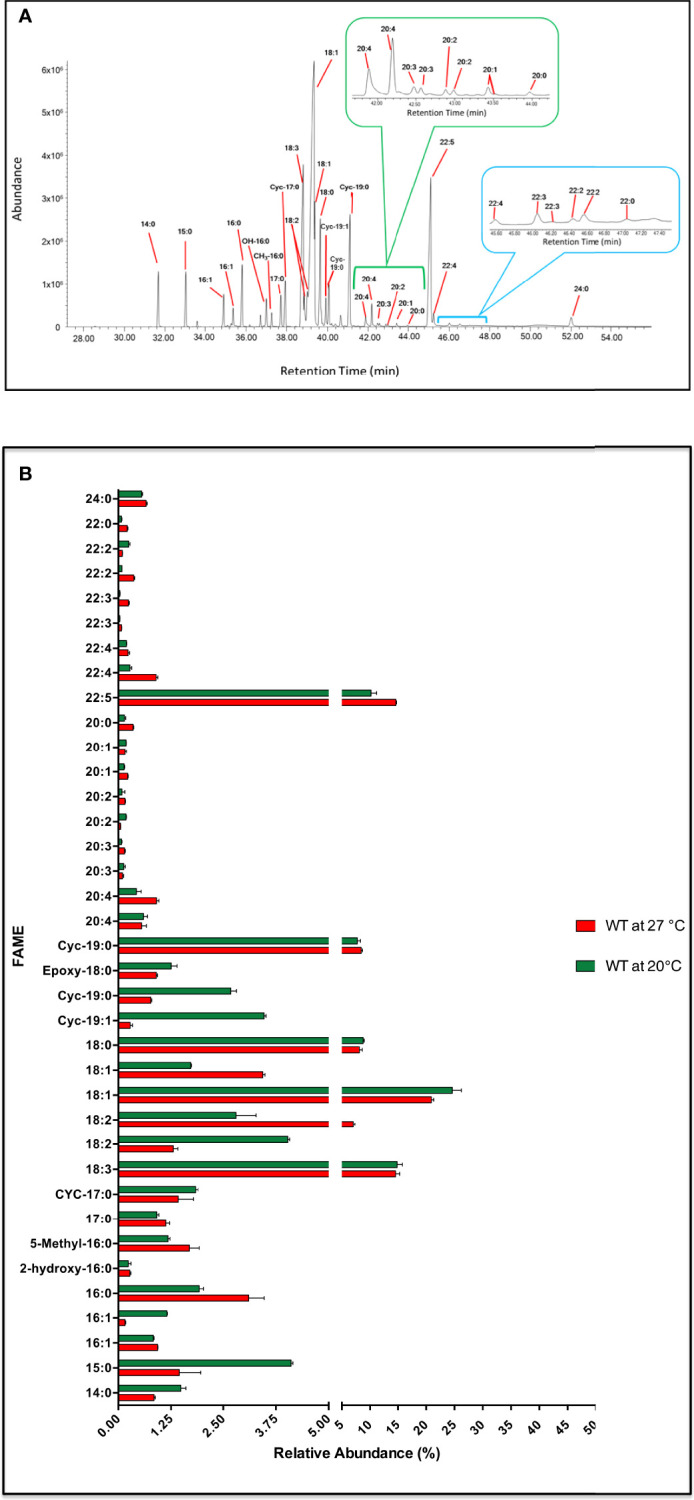
Fatty acid profile in *C. fasiculata*. **(A)** GC-MS chromatogram analysis of WT *C. fasiculata* cultured in standard fat (serum)-free media. Peaks, eluted at different retention times (X axis) and with different abundance (Y axis), are assigned to correspondent FAs. The 20C (green bracket and insert) and 22C PUFAs (light blue bracket and insert) are expanded. **(B)** The bar chart shows the FAME (or FAs) (Y axis, the order follows increasing retention time) and the relative abundance (X axis) found in *C. fasiculata* WT grown in standard fat (serum)-free media at 27°C and at 20°C, as shown in the legend. Values are the mean of three independent biological replicates (n=3). Error bars represent the standard deviation of each mean (±). All FAs were identified using GC-MS based upon retention time, fragmentation, and comparison with standards. Statistical analysis was performed by PRISM 6 by using One-way ANOVA multiple comparisons based on a Tukey t-test with a 95% confidence interval.

Here we report an extensive GC-MS analysis of the large variety of FAs that *C. fasciculata* synthesise when cultured in standard fat (serum)-free media at 27°C and 20°C (**Figure 7A** and **Figure 7B**). Short and medium chain fatty acids such as myristic acid (14:0, rt = 33.02 min), pentadecanoic acid (15:0, rt = 33.02 min), palmitic acid (16:0, rt = 35.81 min), margaric acid (17:0, rt = 37.26 min) and stearic acid (18:0) (rt = 39.65 min, ~ 8%) were identified upon both conditions (**Figures 7A, B**; **Table S12**). Particularly, 14:0 and 15:0 were synthesised at higher level, whereas 16:0 at lower level, at lower temperature (**Table S12**). Two isomers of 16:1 (rt = 35.37 and 34.91 min) were also identified, with low levels of branched and hydroxylated FAs, derived from 16:1, i.e. hydroxy-16:0 (rt = 36.72 min) and methyl-16:0 (rt = 37.00 min) (**Figures 7A, B**; **Table S12**). The most abundant FA was found to be 18:1 (rt = 39.34 min), with a relative abundance of ~ 21% at 27°C and ~ 24% at 20°C. Moreover, a second isomer of 18:1 (rt = 39.35 min) was also identified, but only at 3.44% at 27°C and ~ 1.7% at 20°C (**Figures 7A, B**; **Table S12**). Two species of 18:2 (rt = 38.86 min and 39.01 min) were also identified, accounting for ~ 8% of the total FA content, despite being oppositely increased/decreased by varying the temperature (**Table S12**). The second most abundant FA was 18:3 (rt = 38.81 min) with a relative abundance of 15% upon both higher and lower temperature. Some rarely occurring cyclopropyl-FAs, such as two species of 19:0 Δ (rt = 40.07 and 41.1 min, ~ 9%), a 19:1 Δ (rt = 39.92 min, 0.3-3.5%) and a 17:0 Δ (rt = 37.72 min, ~ 1.5%) were also detected. Interestingly, 19:1 Δ and 19:0 Δ were produced in larger amount at 20°C (**Figures 7A**, **B**; **Table S13**).

The presence of various 20C polyunsaturated fatty acids (PUFAs) (accounting for ~2.5% of the total) were observed eluting between 41.9 - 43.95 min (**Figures 7A, B**; **Table S13**). Amongst those were two species of 20:4 (rt = 41.9 and 42.2 min), 20:3 (rt = 42.29 and 42.49 min), 20:2 (rt = 42.59 and 42.97 min) and 20:1 (rt = 43.42 and 43.45 min). 22C PUFAs were detected at much higher levels, accounting for 16% at 27˚C and 11% at 20°C of the total, with the most abundant being 22:5 (rt = 45.09 min, relative abundance of ~15%). Two species of 22:4 (rt = 45.23 and 46.01 min), 22:3 (rt = 46.18 and 46.41 min) and 22:2 (rt = 46.52 and 47.01 min) were also identified. Long chain (saturated fatty acid) SAFAs, such as behenic acid (22:0, rt = 47.15 min) and lignoceric acid (24:0, rt = 52.00 min) were also observed, but at low abundance (**Figures 7A**, **B**; **Table S13**).

#### Testing the plasticity of *C. fasciculata* fatty acids metabolism: by supplementation with cheap commercial oils and sugar sources

**Figure 8:**
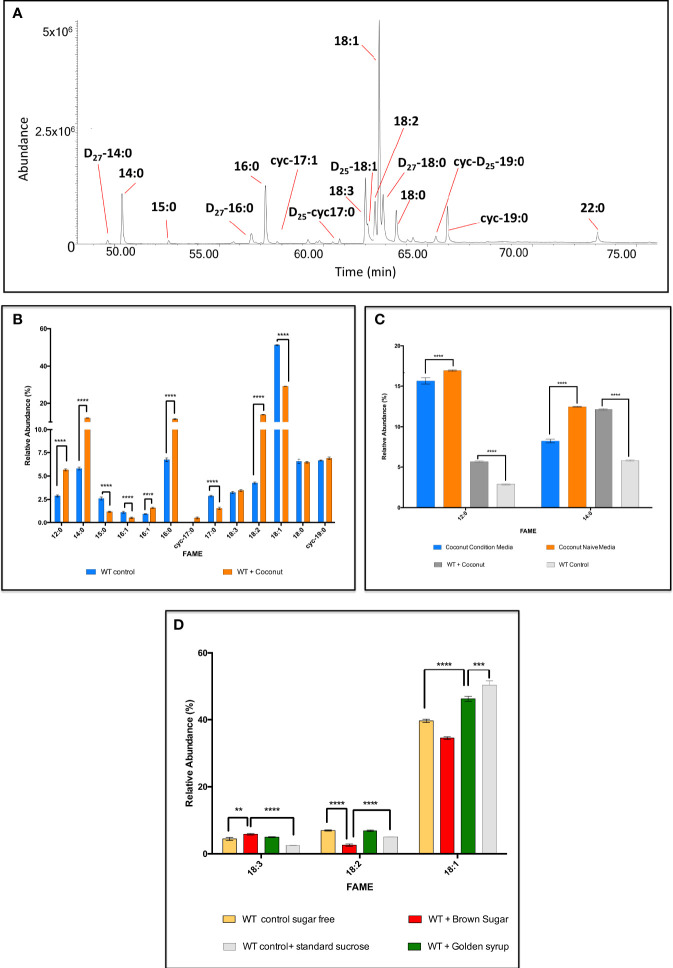
GC-MS analysis of the fatty acids synthesised by *C. fasiculata* WT upon supplementation with various fatty acid and carbohydrate sources. **(A)** GC-MS chromatogram of the FA profile after internalisation of D_27-_myristic acid by **C. fasiculata*.* The cells were supplemented with 50 μM of D_27-_myristic acid in minimal media (absence of Tween-80). The parasites showed not only to be able to internalise D_27-_myristic acid, but also to use it as alternative building block to produce various deuterated FAs. **(B)** GC-MS analysis of the fatty acids in *C. fasiculata* grown in minimal media supplemented with coconut oil. The bar chart shows the FA (X axis) profile and the relative abundance (Y axis) found in *C. fasiculata* chemically supplemented with coconut oil and the WT control, as shown in the legend. **(C)** GC-MS analysis of the fatty acids in the naïve and conditioned media from *C. fasiculata* cells supplemented with coconut oil. The bar chart shows the variation of the relative abundance (Y axis) of 12:0 and 14:0 (X axis) found in the media immediately after supplementation with coconut oil (naïve media), and after 48 h incubation (condition media). Relative abundances of 12:0 and 14:0 are also reported for WT control and WT supplemented with coconut media to highlight their level of internalisation and consumption. **(D)** GC-MS analysis of the fatty acids in *C. fasiculata* grown in minimal media supplemented with brown sugar and golden syrup. The bar chart shows 18C UFAs (X axis, the order follows increasing retention time) and their relative abundance (Y axis) found in *C. fasiculata* chemically supplemented with brown sugar and golden syrup and compared to WT control grown in sugar-free media or with standard sucrose, as shown in the legend. Values are the mean of three independent biological replicates (n=3). Error bars represent the standard deviation of each mean (±). All FAs were identified using GC-MS based upon retention time, fragmentation, and comparison with standards. Statistical analysis was performed by PRISIM 6 by using One-way ANOVA multiple comparisons based on a Tukey t-test with a 95% confidence interval. **** is p ≤ 0.0001, *** is p ≤ 0.001, ** is p ≤ 0.01 and * is p ≤ 0.05.

A series of explorative experiments were conducted after modifying the composition of the media, by removing and/or changing type and concentration of the carbon sources available to the cell. This allowed a preliminary study around the adaptability of FA metabolism in *C. fasciculata* after chemical manipulation of the media. Initially, Tween-80, with its FA-like structure, was removed from the media, to reduce any external lipid-like C-source available to the cells. Initially, Tween-80 was replaced with 50 μM of fully deuterium-labelled myristic acid, D_27_-14:0 (**Figure 8A**). After 48 h at 27°C, *C. fasciculata* had internalised a significant amount of the D_27_-myristic acid and used as a substrate for the elongation and desaturation of FAs (**Figure 8A**). GC-MS analysis revealed the presence of D_27_-palmitic acid (D_27_-16:0) and D_27_-stearic acid (D_27_-18:0). D_25_-18:1 was the only deuterated and unsaturated species identified, where the addition of the double bond had occurred *via* a process of deuterium elimination. Furthermore, D_25_-17:0Δ and D_25_-19:0Δ were identified as cyclic FAs derived from D_25_-16:1 and D_25_-18:1 (**Figure 8A**).

This clearly showed that *C. fasciculata* are able to take up and utilise to the presence of an alternative and fatty acid source. Taking advantage of this high adaptability, the use of alternative and cheap sources of fatty acids was explored. Firstly, small amounts of commercial oils, such as used sunflower oil, sunflower oil, olive oil, rapeseed oil, sesame oil, walnut oil, coconut oil, grape seed oil, were selected to supplement minimal media (containing only minimal Tween-80 to allow homogeneous emulsification). After 48 h at 27°C, the parasites showed to be able to grow and divide, although maintaining a lower cell density, compared to the WT (**Figure S4**). This suggests a rapid adaptation to the unusual conditions in the culture media. Further confirmation was given by GC-MS analysis, which highlighted fully adapted and active FA metabolism (**Figure S5**). Promisingly, cells supplemented with coconut oil showed the best results. Particularly, dodecanoic acid (12:0), 14:0, 16:0 and 18:2 were respectively synthesised at 1.9-fold, 2.1-fold, 1.7-fold and 3.2-fold greater levels in the supplemented cells (p < 0.0001) (**Figure 8B**; **Table S13**). Other FAs, decreased in relative abundance, i.e. 15:0, the other isomer of 16:1, 17:0, and 18:1 were respectively reduced by 2.2-fold, 2.0-fold, 1.9-fold and 1.8-fold (p < 0.0001 for all) (**Figure 8B**; **Table S13**). Additionally, the FA profile of the culture media, immediately after the oil addition (naïve media) and at the spent media (condition media), both for supplemented cells and WT control, were analysed and compared (**Figure 8C**; **Table S13**). This gave an insight on the efficiency of the internalisation and utilisation of the oil components by the parasites. The cells internalised most of the FAs contained in the oil, and efficiently metabolised them into longer SAFAs and PUFAs. Particularly, the most significant of which was the uptake and elongation of the most abundant components of coconut oil: 12:0 and 14:0 (**Figure 8C**; **Table S13**). These data suggested that the cells were able to internalise ~1.3% of 12:0 and ~ 3.9% of 14:0 to produce higher amounts of monounsaturated fatty acids (MUFAs) and PUFAs resulting in the higher production of 18:2 previously described (~ 9.7% higher than the wild type) (**Figure 8C**, **Table S13**). After showing that *C. fasciculata* were able to produce PUFAs from oils containing complex mixtures of SAFAs and MUFAs,

To explore *C. fasciculata* fatty acid/lipid metabolism versatility further, the parasites were supplemented with four different commercial sugars: brown sugar, maple syrup, golden syrup, corn syrup, in the absence of any lipid/fatty acid source. A minimal culture media was used to push the metabolic adaptation of the cells even further: Tween-80 and sucrose were completely removed from the media. After 48 h at 27°C, the growth rate of the sugar supplemented parasites was found higher than the WT control, cultured in the sugar-free media. Furthermore, the cell density was found to be greater upon sugar supplementation than in the media containing the same amount of commercial cell culture sucrose (**Figure S6**). The analysis of the FAs *via* GC-MS, both in complete absence of carbohydrate sources, and in presence of alternative ones, displayed the ability of the cells of producing various species of FAs (**Figure S7**; **Table S14**). Brown sugar and golden syrup showed to be the best C-sources: higher amount of 18C UFAs were detected (**Figure 8D**, **Table S14**). Particularly, 18:3 was found to be 1.3-fold (p = 0.0014) higher in the parasites supplemented with brown sugar than in the sugar free media (**Figure 8D**; **Table S14**). Surprisingly, a 2.3-fold (p < 0.0001) increase was detected against the cells grown with the media containing the same amount of standard sucrose. Consequently, 18:2 was reduced by 2.7-fold (p < 0.0001) and 1.4-fold (p = 0.0002) in the cells supplemented with brown sugar in the same comparisons. The supplementation with golden syrup determined an increase of 1.2-fold in 18:1 compared to WT grown in sugar-free media (p < 0.0001). On the other hand, a 1.1-fold (p = 0.0001) decrease was revealed against WT cells grown with the standard culturing sucrose (**Figure 8D**; **Table S14**). The results obtained after chemical supplementation and manipulation of the media showed that *C. fasciculata* can be considered as a highly performing and easily tuneable bio-system for low-cost production of PUFAs. In fact, by using cheap and commonly available sugar sources, which are non-FA sources, and cooking oils, containing instead mixture of different FAs, these parasites were able to produce large quantities of diverse FAs.

## Discussion

As a rapidly dividing eukaryote, it is to be expected that *Crithidia* possess a high level of lipid biosynthesis to accommodate a need for new membranes.

Based upon the al approach of extended genome database mining, combined with the lipidomic analyses, it is clear that *Crithidia* are able to biosynthesise all of the main phospholipid species that would be expected from eukaryotes, including: PC, PE, PI, PS, PG, CL and PIPs. Additionally, *Crithidia* exclusively produce IPC as their main sphingolipid species, suggesting that their sphingolipid metabolism/biosynthesis is closer to that of *Leishmania* and *T. cruzi* rather than *T. brucei (* Smith and Bütikofer, 2010; Zhang and Beverley, 2010; Booth and Smith, 2020 *).*

High abundance of a cyclopropyl fatty acid was identified within *Crithidia* extracts, which is unusual as the production of these species is more commonly associated with prokaryotes (Grogan and Cronan, 1997). In addition to *Crithidia*, other kinetoplasts known to produce this C19Δ fatty acid are: *L. infantum*, *L. braziliensis* and *L. mexicana*, but absence in *L. major* due to the loss of the cyclopropyl fatty acid synthase gene. It has been recently shown that the production of this cylcopropyl fatty acids is a virulence factor in *L. infantum* (Oyola et al., 2012). However, as *Crithidia* are a non-pathogenic species, it is more likely that this C19Δ fatty acid is used in a membrane structural role showing resistance to environmental stresses such as pH, ionic strength and temperature as is the case for bacteria, that also have high levels of this cyclopropyl fatty acid at late log phase. Further investigation revealed that *Crithidia* utilise this fatty acid in the production of several PE species, again showing a similarity to *L. infantum*, which can produce C19Δ containing PE species.

Remarkably *Crithidia* showed sustained growth within a fatty acid deficient medium. It was also shown through labellings with deuterated myristic acid, that *Crithidia* can clearly uptake and incorporate extracellular fatty acids into their lipid pools, thus highlighting the possible activity of a fatty acid transporter. This is made possible by the vast repertoire of tuneable destaurases and elongases encoded in *C. fasciculata*’s genome. In fact, these enzymes revealed the ability to work in a concerted and sequential manner along the biocatalytic pathway to produce SAFAs, MUFAs and PUFAs using the sources available to them. Moreover, *C. fasciculata* revealed the ability of producing rarely occurring FAs in eukaryotes, such as cyclopropyl, hydroxyl and branched SAFAs, which underlined that other enzymes (CFAS, FAs hydroxylase etc) may also intervene to improve the FAs molecular diversity even further (Einicker-Lamas et al., 2007). These FAs, and particularly 17:0 Δ and 19:0 Δ, had been previously demonstrated to be very important in process such membrane fluidity maintenance, during cell adaptation to the environment. This was clearly shown to be very efficient in this study too (Concepcion et al., 1998). Importantly, *C. fasciculata* were able to produce *de novo* large amounts of ω-3 PUFAs such as α-linolenic acid (Δ-18:3 (Meyer and Holz, 1966; Ferguson, 1997; MacKenzie et al., 2009)) and docosapentaenoic acid (Δ-22:5 (Sotirhos et al., 1986; Ferguson, 1997; Martin and Smith, 2006a; Smith and Bütikofer, 2010; Aslett et al., 2010)) which are EPUFAs involved in numerous biological and physiological pathways in higher eukaryotes, such as mammals (Kidder and Dutta, 1958). Furthermore, *C. fasciculata* displayed high adaptability to the most various fats and carbon sources contained in the culture media, such as pure fatty acids, complex mixture of those in cheap cooking oils, and carbohydrates from commercial sugars. In these unusual environments, the parasites continued to grow rapidly, to produce large amounts of biomass from small volume of culture, to scavenge any of the available fatty acid and sugar sources from the media, and to adapt/tune their UFAs and PUFAs metabolism by up- or down-regulating the activity of elongases and desaturases.

Building upon this evidence, the potential of increasing the production of high value PUFAs, by exploiting the promising metabolic ‘expertise’ of *C. fasciculata*, is the future scope of our future studies in this area. This could offer a valid alternative in response to the current shortage of dietary ω-3 and ω-6 EPUFAs for humans and animals, caused by the agricultural and industrial revolutions and climate change (Acosta-Serrano et al., 2001). Particularly, this situation has dramatically affected the marine ecosystem, which is currently one of the main PUFAs producers. Moreover, vegetable oil plants, which are currently the most exploited of FA sources, did not seem to be able to meet the constantly increasing PUFAs demand worldwide (Dostálová and Volf, 2012). On the other hand, FAs chemical synthesis is extremely challenging and time consuming, whereas the latest biotechnological advances for PUFAs production are often too expensive (Smith, 2015; Cerone and Smith, 2021). Thus, *C. fasciculata* might represent an innovative and alternative microbial system by which one can access ω-3 PUFAs in a larger amount and in a more sustainable way.

In should be noted of course that the use of tainted energy sources, such as used cooking oils may potential contain potential harmful toxins, such as oxidised lipids/fatty acids, that could potentially be detrimental for cellular growth, but also potentially utilised as intermediates in beta-oxidation of fatty acids. The growth conditions that have been utilised to grow *C. fasciculata in vitro* are obviously removed from those experienced *in vivo* by both the choanomastigote and amastigote forms of this kinetoplastid. However, *C. fasciculata* and their opportunistic metabolism have likely evolved from their *in vivo* life

## Methods

### Crithidia fasciculata

*C. fasciculata* clone HS6 were grown at pH 7.6 in buffered serum-free medium containing 5g/L of yeast extract, 4 g/L of tryptone, with or without 15 g/L or 1.5 g/L of sucrose, 4.4 g/L of triethanolamine hydrochloride, with or without 0.5% v/v or 0.05% v/v of Tween-80, 10 μg/mL of haemin, 71.5 g/L of HEPES. Cells were incubated at 27°C with gentle agitation, maintained at mid-log phase and passaged every 3 days (Scolaro et al., 2005). Cell counting was performed using a haemocytometer.

### Chemical supplementation of *Crithidia fasciculata* culture media with deuterium labelled fatty acid

D_27_-myristic acid was conjugated with defatted bovine serum albumin (BSA) in a 5% aqueous solution. Initially 50 mM D_27_-myristic acid stock solution (stock solution A) was prepared in absolute EtOH. 1 mM of D_27_-myristic acid stock solution (stock solution B) was prepared by diluting stock solution A in 5% defatted BSA solution. The buffered serum-free minimal media (no Tween-80) was supplemented by adding stock solution B to the culture media to final concentrations 50 μM of D_27_-myristic acid and 0.25% defatted BSA into the final cell culture volume.

#### Chemical supplementation of *Crithidia fasciculata* culture media with commercial oils and sugars

20 mL (the volume of media is for each cell culture sample) of buffered serum-free minimal media with 10% Tween-80 and 10% sucrose were supplemented with 150 μL of various oils (sesame oil, coconut oil, walnut oil, grapeseed oil, rapeseed oil, sunflower oil, used sunflower oil, olive oil). 20 mL of buffered serum-free minimal media without Tween-80 and sucrose were supplemented with 750 μg of various sugars (brown sugar, golden syrup, maple syrup, corn syrup, sucrose).

#### Growth curve for *Crithidia fasciculata*

*C. fasciculata* cells were grown to mid-log phase in standard serum-free media, or in minimal media with 10% or no Tween-80, and with 10% or no sucrose, with or without external sources supplementation, and distributed into non-vented flask at an equal density of 5 x 10^4^ cell/mL. The cells were grown for 48 h or 72 h and counted using a haemocytometer every 24 h.

#### Lipid extraction

Mid log phase *C. fasciculata* (~ 2x10^7^ cell/mL) were collected by centrifugation at 800 x *g* for 10 min. The cell pellet was re-suspended in a minimal quantity of media (~ 500 μL) and transferred to a microfuge tube for further centrifugation at 3800 x *g.* Cells were washed respectively with PBS, re-suspended in 100 μL of PBS and transferred to a glass vial. 375 μL of 2:1 (v/v) of MeOH : CHCl_3_ solution were added for biphasic separation based on the followed method described by Bligh-Dyer (Bligh and Dyer, 1959), The organic phase is dried under nitrogen gas stream and stored in glass vials at 4°C until further analysis or experiments.

#### Fatty acids transmethylation and gas chromatography – mass spectroscopy analysis

Acid hydrolysis of free fatty acids was performed on the dry total lipid extracts. The reaction (total volume 1mL) is conducted in a glass vial. 100 μL of toluene were added, followed by 750 μL of MeOH and 150 150 μLof 8% HCl MeOH:H_2_O 85:15 (v/v) solution in order to allow the esterification of the free fatty acids. The reaction is left to go to completion at 45°C overnight. Upon drying, the fatty acid methyl esters (FAMEs) were extracted with a 1:1 hexane:H_2_O. The FAME extracts were dried under nitrogen gas stream. The FAME extracts were dissolved in dichloromethane, typically 20 μL and 1 μL is analysed by GC-MS on an Agilent Technologies GC-6890N gas chromatograph coupled to an MS detector‐5973. Separation by GC was performed using a PhenomenexZB-5 column (30 M x 25 mm x 25 mm), with a temperature program of 70°C for 10 min, followed by a gradient to 220°C, at 5°C/min and maintained at 220°C for a further 15 min. Mass spectra were acquired from 50-500 amu. The identity of FAMEs was carried out by comparison of the retention time and fragmentation pattern against bacterial and mammalian FAME standards and online available FAME library (http://www.lipidhome.co.uk/ms/methesters/me-arch/index.htm).

#### Electrospray ionization tandem mass spectrometry

Lipid samples were analysed by electrospray ionization tandem MS (ESI-MS/MS) with an AB-Sciex Qtrap 4000 triple quadrupole mass spectrometer fitted with an Advion TriVersa Nanomatte nanoelectrospray. Survey scans in negative mode was used for the detection of PE, EPC, PI, IPC, PS, PG and PA (cone voltage=1.25 kV). Positive ion mode survey scans were used to detect PC and SM (cone voltage=1.25 kV). In negative ion mode, precursor of m/z 196 scans were used to detect PE and EPC species, precursor of m/z 241 scans for IPC and PI species, precursor of m/z 153 scans for PG and PA, (collision energy, CE = 60 eV). In positive ion mode, precursor of m/z 184 scans were used to detect PC and SM species (CE = 60 eV). Neutral loss of m/z 87 scans for PS (CE = 60 eV). Spectra were acquired over or within a range of 120-1000 m/z, and each spectrum represents a minimum of 30 consecutive scans with nitrogen collision gas. Samples were run using a 1:1 solvent mixture of 2:1(v/v) MeOH : CHCl_3_ and 6:7:2 (v/v) acetonitrile:isopropanol:dH_2_O. Individual lipid species were annotated according to their acyl composition determined by daughter ion scans produced and compared to previous lipid identification by Richmond et al. (2010), to theoretical values contained in the Lipid Metabolites and Pathways Strategy consortium database (LIPID MAPS, http://www.lipidmaps.org/).

#### High-resolution mass spectrometry

Dried lipid extracts (as above) were analysed by the University of St Andrews Mass Spectrometry service ESI-MS upon a Thermo-scientific Exactive mass spectrometer tuned to an accuracy of > 1 ppm. Survey scans were acquired in both the positive and negative ion modes over a range of 80-1600 m/z (or a portion thereof) with a cone voltage of 3.7kV. Each spectrum consists of a minimum of 20 consecutive scans.

#### Genomic analysis

The identification of *Crithidia* genes was conducted utilising datasets contained within the TriTryp database (Aslett et al., 2010). Sequence similarity was determined by the NCBI-BLAST algorithm (Altschul et al., 1990) using the blastp program to search peptide sequences identified by Peacock et al (Peacock et al., 2007), Kabani et al. (2009), Oyola et al. (2012) and Lykidis (2007) against the TriTryp database. All homologs identified had a cut off threshold of greater than 30% similarity, in the cases where the tables show no corresponding *T. brucei* or *Leishmania* homologues these are due to *Crithidia* having multiple homologues within that specific enzyme activity, i.e. acyl-CoA synthatases.

#### NMR

Lipid extracts were prepared as above and placed into 1:1 CDCl_3_:CD_3_OD for analysis (Mwenechanya et al., 2017).P NMR analysis was performed by the University of St Andrews liquid-state NMR service at 25°C upon a Bruker Avance 400 MHz NMR spectrometer with (Kaufer et al., 2017)H-decoupling. Identification of lipid classes was carried out by comparison of each chemical shift to that of lipid standards representing: PG, PA, PI, PE, PS, SM, PC and LPC using FOS-CHO 8 as an internal standard (Sotirhos et al., 1986; Murgia et al., 2003; MacKenzie et al., 2009).

## Data availability statement

The original contributions presented in the study are included in the article/**Supplementary Material**. Further inquiries can be directed to the corresponding author.

## Author contributions

MR, MC, TS all undertake experimentation. TS conceived experiments. MR, MC, TS wrote paper. TS reviewed paper. All authors contributed to the article and approved the submitted version.

## Funding

We would like to thank the Engineering and Physical Sciences Research Council, University of St. Andrews, and the EPSRC Centre for Doctoral Training in Critical Resource Catalysis (CRITICAT) for financial support [Ph.D. studentship to MC; Grant code: EP/L016419/1].

## Acknowledgments

We would like to thank the Criticat Students Conor Oates, Megan Bryden and Chris Thomson who participated in some of the early ground work on the supplementation of *Crithidia* with various oils and sugars.

## Conflict of interest

The authors declare that the research was conducted in the absence of any commercial or financial relationships that could be construed as a potential conflict of interest.

## Publisher’s note

All claims expressed in this article are solely those of the authors and do not necessarily represent those of their affiliated organizations, or those of the publisher, the editors and the reviewers. Any product that may be evaluated in this article, or claim that may be made by its manufacturer, is not guaranteed or endorsed by the publisher.
