# Supplementary material for: The lipidome of Crithidia fasiculataand its plasticity
Source: Front Cell Infect Microbiol. 2022 Oct 28;12:945750. doi: 10.3389/fcimb.2022.945750 (PMC9671073; doi:10.3389/fcimb.2022.945750)
Supplement: Supplementary file 1 [file DataSheet_1.docx]

SUPPLEMENTARY MATERIAL

The Lipidome of Crithidia fasiculata and its plasticity.

Michela Cerone^1^, Mathew Roberts^1^ and Terry K Smith^,1*^

^1^BSRC Schools of Biology and Chemistry, University of St Andrews
North Haugh, St Andrews KY16 9ST, UK

* Correspondence:
Terry K Smith
tks1@st-andrews.ac.uk

A)

*
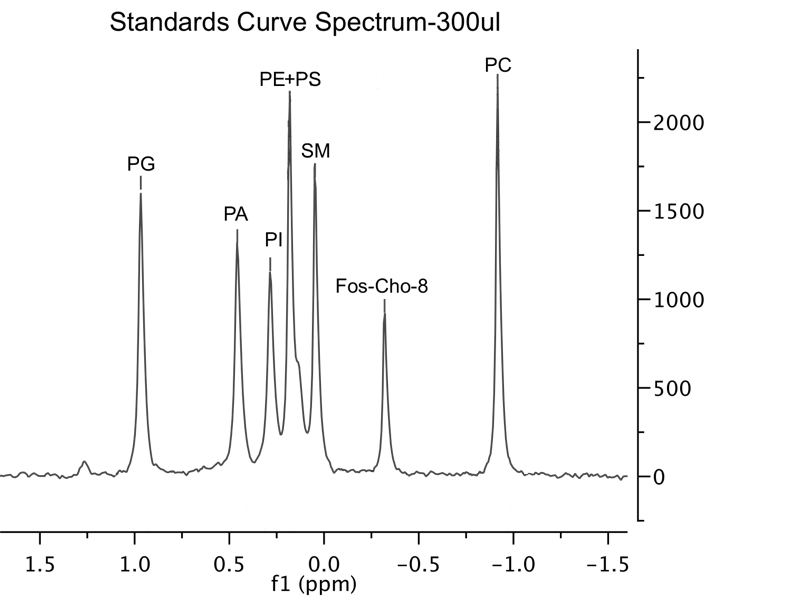
*

B)

*
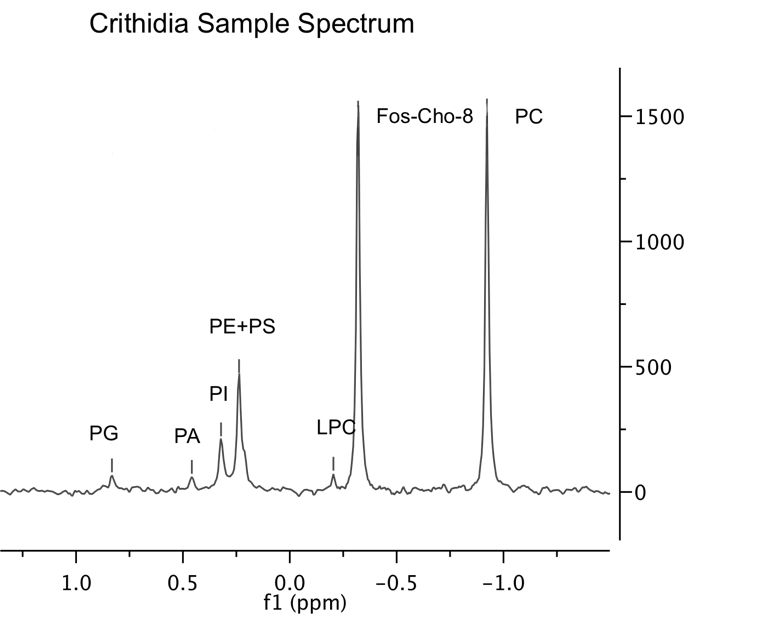
*

Figure S1; ^31^P-NMR analysis of A) Mixture of lipid standards and B) Cellular lipid extract from *Crithidia fasiculata*. The lipid analogue Fos-Cho-8 was used as an internal standard.

Table S1; Assignments of phospholipid species observed in positive ion mode HR-MS

| Peak | Observed HR m/z | Lipid Component | Principle Component | Theoretical m/z |
| --- | --- | --- | --- | --- |
| A |  |  |  |  |
| 1 | 730.5671836 | [PC(32:1)](http://www.lipidmaps.org/tools/ms/G_expand.php?ABBREV=PC%2832:1%29) |  | 730.5392 |
| 2 | 732.5542077 | [PC(32:0)](http://www.lipidmaps.org/tools/ms/G_expand.php?ABBREV=PC%2832:0%29) |  | 732.5549 |
| 3 | 734.6527022 | [PC(P-34:5)](http://www.lipidmaps.org/tools/ms/G_expand.php?ABBREV=PC%28P-34:5%29) |  | 734.513 |
| 4 | 736.4871603 | [PC(O-34:5), PC(P-34:4)](http://www.lipidmaps.org/tools/ms/G_expand.php?ABBREV=PC%28O-34:5%29) |  | 736.5286 |
| 5 | 738.5102195 | [PC(O-34:4), PC(P-34:3)](http://www.lipidmaps.org/tools/ms/G_expand.php?ABBREV=PC%28O-34:4%29) |  | 738.5443 |
| 6 | 740.559548 | [PC(O-34:3), PC(P-34:2)](http://www.lipidmaps.org/tools/ms/G_expand.php?ABBREV=PC%28O-34:3%29) |  | 740.5599 |
|  | 742.5389336 | [PC(34:9)](http://www.lipidmaps.org/tools/ms/G_expand.php?ABBREV=PC%2834:9%29) |  | 742.4453 |
|  | 742.5764276 | [PC(O-34:2), PC(P-34:1)](http://www.lipidmaps.org/tools/ms/G_expand.php?ABBREV=PC%28O-34:2%29) | | 742.5756 |
|  | 744.4959157 | [PC(34:8)](http://www.lipidmaps.org/tools/ms/G_expand.php?ABBREV=PC%2834:8%29) |  | 744.461 |
|  | 744.5566235 | [PC(O-34:1), PC(P-34:0)](http://www.lipidmaps.org/tools/ms/G_expand.php?ABBREV=PC%28O-34:1%29) | | 744.5912 |
|  |  |  |  |  |
| B |  |  |  |  |
| 7 | 746.5547351 | [PC(34:7)](http://www.lipidmaps.org/tools/ms/G_expand.php?ABBREV=PC%2834:7%29) |  | 746.4766 |
| 8 | 746.5547351 | [PC(O-34:0)](http://www.lipidmaps.org/tools/ms/G_expand.php?ABBREV=PC%28O-34:0%29) |  | 746.6069 |
| 9 | 748.484701 | [PC(34:6)](http://www.lipidmaps.org/tools/ms/G_expand.php?ABBREV=PC%2834:6%29) |  | 748.4923 |
| 10 | 750.5006359 | [PC(34:5)](http://www.lipidmaps.org/tools/ms/G_expand.php?ABBREV=PC%2834:5%29) |  | 750.5079 |
|  | 752.5154717 | [PC(34:4)](http://www.lipidmaps.org/tools/ms/G_expand.php?ABBREV=PC%2834:4%29) |  | 752.5236 |
|  | 754.5403755 | [PC(34:3)](http://www.lipidmaps.org/tools/ms/G_expand.php?ABBREV=PC%2834:3%29) |  | 754.5392 |
|  | 756.5555822 | [PC(34:2)](http://www.lipidmaps.org/tools/ms/G_expand.php?ABBREV=PC%2834:2%29) |  | 756.5549 |
|  |  |  |  |  |
| C |  |  |  |  |
| 11 | 758.5699406 | [PC(34:2)](http://www.lipidmaps.org/tools/ms/G_expand.php?ABBREV=PC%2834:2%29) |  | 758.5695 |
| 12 | 760.585266 | [PC(34:1)](http://www.lipidmaps.org/tools/ms/G_expand.php?ABBREV=PC%2834:1%29) |  | 760.5851 |
| 13 | 762.1 | PC 34:0 | PC 16:0/18:0 |  |
| 14 | 762.502336 | [PC(P-36:5)](http://www.lipidmaps.org/tools/ms/G_expand.php?ABBREV=PC%28P-36:5%29) |  | 762.5443 |
|  | 764.5175277 | [PC(O-36:5), PC(P-36:4)](http://www.lipidmaps.org/tools/ms/G_expand.php?ABBREV=PC%28O-36:5%29) | | 764.5599 |
|  | 766.517567 | [PC(O-36:4), PC(P-36:3)](http://www.lipidmaps.org/tools/ms/G_expand.php?ABBREV=PC%28O-36:4%29) | PC p-18:0/18:3 | 766.5756 |
|  | 768.5144622 | [PC(36:10)](http://www.lipidmaps.org/tools/ms/G_expand.php?ABBREV=PC%2836:10%29) |  | 768.461 |
|  | 768.5911372 | [PC(O-36:3), PC(P-36:2)](http://www.lipidmaps.org/tools/ms/G_expand.php?ABBREV=PC%28O-36:3%29) | | 768.5912 |
|  | 770.5054402 | [PC(36:9)](http://www.lipidmaps.org/tools/ms/G_expand.php?ABBREV=PC%2836:9%29) |  | 770.4766 |
|  | 770.5701211 | [PC(O-36:2), PC(P-36:1)](http://www.lipidmaps.org/tools/ms/G_expand.php?ABBREV=PC%28O-36:2%29) | | 770.6069 |
|  |  |  |  |  |
| D |  |  |  |  |
| 15 | 772.4875632 | [PC(36:8)](http://www.lipidmaps.org/tools/ms/G_expand.php?ABBREV=PC%2836:8%29) |  | 772.4923 |
| 16 | 772.5864064 | [PC(O-36:1), PC(P-36:0)](http://www.lipidmaps.org/tools/ms/G_expand.php?ABBREV=PC%28O-36:1%29) | | 772.6225 |
| 17 | 774.5019872 | [PC(36:7)](http://www.lipidmaps.org/tools/ms/G_expand.php?ABBREV=PC%2836:7%29) |  | 774.5079 |
|  | 774.5916383 | [PC(O-36:0)](http://www.lipidmaps.org/tools/ms/G_expand.php?ABBREV=PC%28O-36:0%29) |  | 774.6382 |
|  | 776.5156652 | [PC(36:6)](http://www.lipidmaps.org/tools/ms/G_expand.php?ABBREV=PC%2836:6%29) | PC 14:0/22:6 | 776.5236 |
|  | 780.5311583 | [PC(36:4)](http://www.lipidmaps.org/tools/ms/G_expand.php?ABBREV=PC%2836:4%29) |  | 780.5549 |
|  | 782.5696718 | [PC(36:3)](http://www.lipidmaps.org/tools/ms/G_expand.php?ABBREV=PC%2836:3%29) |  | 782.5705 |
|  |  |  |  |  |
| E |  |  |  |  |
| 18 | 784.5543875 | [PC(36:2)](http://www.lipidmaps.org/tools/ms/G_expand.php?ABBREV=PC%2836:2%29) | PC 18:1/18:1 | 784.5862 |
| 19 | 786.5190174 | [PC(36:1)](http://www.lipidmaps.org/tools/ms/G_expand.php?ABBREV=PC%2836:1%29) |  | 786.6018 |
| 20 | 786.5667425 | [PC(36:1)](http://www.lipidmaps.org/tools/ms/G_expand.php?ABBREV=PC%2836:1%29) |  | 786.6018 |
|  | 789.7 | [PC(36:0)](http://www.lipidmaps.org/tools/ms/G_expand.php?ABBREV=PC%2836:2%29) | PC 14:0/22:0 & PC 16:0/20:0 |  |
|  | 790.566426 | [PC(O-38:6), PC(P-38:5)](http://www.lipidmaps.org/tools/ms/G_expand.php?ABBREV=PC%28O-38:6%29) |  | 790.5756 |
|  | 792.5316314 | [PC(O-38:5), PC(P-38:4)](http://www.lipidmaps.org/tools/ms/G_expand.php?ABBREV=PC%28O-38:5%29) |  | 792.5912 |
|  | 794.5186555 | [PC(O-38:4), PC(P-38:3)](http://www.lipidmaps.org/tools/ms/G_expand.php?ABBREV=PC%28O-38:4%29) |  | 794.6069 |
|  | 796.5457723 | [PC(38:10)](http://www.lipidmaps.org/tools/ms/G_expand.php?ABBREV=PC%2838:10%29) | | 796.4923 |
|  | 796.5457723 | [PC(O-38:3), PC(P-38:2)](http://www.lipidmaps.org/tools/ms/G_expand.php?ABBREV=PC%28O-38:3%29) | | 796.6225 |
|  | 798.5372204 | [PC(38:9)](http://www.lipidmaps.org/tools/ms/G_expand.php?ABBREV=PC%2838:9%29) | | 798.5079 |
|  | 798.5372204 | [PC(O-38:2), PC(P-38:1)](http://www.lipidmaps.org/tools/ms/G_expand.php?ABBREV=PC%28O-38:2%29) |  | 798.6382 |
|  |  |  | |  |
| F |  |  |  |  |
| 21 | 800.5846771 | [PC(38:8)](http://www.lipidmaps.org/tools/ms/G_expand.php?ABBREV=PC%2838:8%29) | | 800.5236 |
|  | 800.5846771 | [PC(O-38:1), PC(P-38:0)](http://www.lipidmaps.org/tools/ms/G_expand.php?ABBREV=PC%28O-38:1%29) | 800.6538 |  |
|  | 802.5703329 | [PC(38:7)](http://www.lipidmaps.org/tools/ms/G_expand.php?ABBREV=PC%2838:7%29) |  | 802.5392 |
|  | 802.5703329 | [PC(O-38:0)](http://www.lipidmaps.org/tools/ms/G_expand.php?ABBREV=PC%28O-38:0%29) |  | 802.6695 |
|  | 804.5772529 | [PC(38:6)](http://www.lipidmaps.org/tools/ms/G_expand.php?ABBREV=PC%2838:6%29) | |  |
|  |  |  |  |  |
| G |  |  |  |  |
| 22 | 830.5909496 | [PC(40:7)](http://www.lipidmaps.org/tools/ms/G_expand.php?ABBREV=PC%2840:7%29) |  | 830.5705 |
|  | 830.5909496 | [PC(O-40:0)](http://www.lipidmaps.org/tools/ms/G_expand.php?ABBREV=PC%28O-40:0%29) |  | 830.7008 |
|  | 832.5336056 | [PC(40:6)](http://www.lipidmaps.org/tools/ms/G_expand.php?ABBREV=PC%2840:6%29) |  | 832.5862 |
|  | 834.5255498 | [PC(40:5)](http://www.lipidmaps.org/tools/ms/G_expand.php?ABBREV=PC%2840:5%29) | PC 18:0/22:5 | 834.6018 |
|  | 836.5367826 | [PC(40:4)](http://www.lipidmaps.org/tools/ms/G_expand.php?ABBREV=PC%2840:4%29) |  | 836.6175 |
|  | 838.544065 | [PC(40:3)](http://www.lipidmaps.org/tools/ms/G_expand.php?ABBREV=PC%2840:3%29) |  | 838.6331 |
|  | 840.5502944 | [PC(40:2)](http://www.lipidmaps.org/tools/ms/G_expand.php?ABBREV=PC%2840:2%29) |  | 840.6488 |
|  |  |  |  |  |
| H |  |  |  |  |
| 23 | 840.5502944 | [PC(40:2)](http://www.lipidmaps.org/tools/ms/G_expand.php?ABBREV=PC%2840:2%29) |  | 840.6488 |
|  | 842.5458527 | [PC(P-42:7)](http://www.lipidmaps.org/tools/ms/G_expand.php?ABBREV=PC%28P-42:7%29) |  | 842.6069 |
|  | 842.5458527 | [PC(40:1)](http://www.lipidmaps.org/tools/ms/G_expand.php?ABBREV=PC%2840:1%29) |  | 842.6644 |
|  | 848.5744894 | [PC(42:12)](http://www.lipidmaps.org/tools/ms/G_expand.php?ABBREV=PC%2842:12%29) |  | 848.5236 |
|  | 848.5744894 | [PC(O-42:5), PC(P-42:4)](http://www.lipidmaps.org/tools/ms/G_expand.php?ABBREV=PC%28O-42:5%29) |  | 848.6538 |
|  | 850.5546766 | [PC(42:11)](http://www.lipidmaps.org/tools/ms/G_expand.php?ABBREV=PC%2842:11%29) | *PC 20:6/22:5* | 850.5392 |
|  | 850.6200373 | [PC(O-42:4), PC(P-42:3)](http://www.lipidmaps.org/tools/ms/G_expand.php?ABBREV=PC%28O-42:4%29) |  | 850.6695 |
| I |  |  |  |  |
| 24 | 878.5854529 | [PC(44:11), PC 40:1 + Cl](http://www.lipidmaps.org/tools/ms/G_expand.php?ABBREV=PC%2844:11%29) | |  |
| 25 | 878.5854529 | [PC(O-44:4), PC(P-44:3)](http://www.lipidmaps.org/tools/ms/G_expand.php?ABBREV=PC%28O-44:4%29) |  | 878.7008 |
| 26 | 880.5649611 | [PC(44:10)](http://www.lipidmaps.org/tools/ms/G_expand.php?ABBREV=PC%2844:10%29) |  | 880.5862 |
| 27 | 880.5649611 | [PC(O-44:3), PC(P-44:2)](http://www.lipidmaps.org/tools/ms/G_expand.php?ABBREV=PC%28O-44:3%29) |  | 880.7164 |
|  |  |  | |  |
| J |  |  |  |  |
|  | 906.6528332 | [PC(46:11)](http://www.lipidmaps.org/tools/ms/G_expand.php?ABBREV=PC%2846:11%29) | | 906.6018 |
|  | 906.6528332 | [PC(O-46:4), PC(P-46:3)](http://www.lipidmaps.org/tools/ms/G_expand.php?ABBREV=PC%28O-46:4%29) |  | 906.7321 |
|  | 908.6558091 | [PC(46:10)](http://www.lipidmaps.org/tools/ms/G_expand.php?ABBREV=PC%2846:10%29) |  |  |
|  | 908.6558091 | [PC(O-46:3), PC(P-46:2)](http://www.lipidmaps.org/tools/ms/G_expand.php?ABBREV=PC%28O-46:3%29) |  |  |
| 28 | 916.6024292 | [PC(46:6)](http://www.lipidmaps.org/tools/ms/G_expand.php?ABBREV=PC%2846:6%29) |  |  |
| 29 | 912.3 |  |  |  |
| K |  |  |  |  |
| 30 | 920.6685204 | [PC(46:4)](http://www.lipidmaps.org/tools/ms/G_expand.php?ABBREV=PC%2846:4%29) | | 920.7114 |
|  | 924.8 | [PC(O-48:5)](http://www.lipidmaps.org/tools/ms/G_expand.php?ABBREV=PC%28O-48:5%29) |  |  |
|  | 930.4776767 | [PC(48:13)](http://www.lipidmaps.org/tools/ms/G_expand.php?ABBREV=PC%2848:13%29) |  | 930.6018 |
|  | 930.4776767 | [PC(O-48:6), PC(P-48:5)](http://www.lipidmaps.org/tools/ms/G_expand.php?ABBREV=PC%28O-48:6%29) |  |  |
| L |  |  |  |  |
| 31 | 932.6671766 | [PC(48:12)](http://www.lipidmaps.org/tools/ms/G_expand.php?ABBREV=PC%2848:12%29) |  | 932.6175 |
| 32 | 932.7485882 | [PC(O-48:5), PC(P-48:4)](http://www.lipidmaps.org/tools/ms/G_expand.php?ABBREV=PC%28O-48:5%29) |  | 932.7477 |
| 33 | 934.6852098 | [PC(48:11)](http://www.lipidmaps.org/tools/ms/G_expand.php?ABBREV=PC%2848:11%29) | |  |
|  | 934.6852098 | [PC(O-48:4), PC(P-48:3)](http://www.lipidmaps.org/tools/ms/G_expand.php?ABBREV=PC%28O-48:4%29) |  | 934.7634 |
|  | 936.6907308 | [PC(48:10)](http://www.lipidmaps.org/tools/ms/G_expand.php?ABBREV=PC%2848:10%29) |  | 934.6331 |
|  | 936.6907308 | [PC(O-48:3), PC(P-48:2)](http://www.lipidmaps.org/tools/ms/G_expand.php?ABBREV=PC%28O-48:3%29) |  |  |
|  | 940.7345293 | [PC(48:8)](http://www.lipidmaps.org/tools/ms/G_expand.php?ABBREV=PC%2848:8%29) | | 936.6488 |
|  | 940.7345293 | [PC(O-48:1), PC(P-48:0)](http://www.lipidmaps.org/tools/ms/G_expand.php?ABBREV=PC%28O-48:1%29) |  |  |

Table S2; Assignments of phospholipid species observed in positive ion mode HR-MS

| Peak | Lipid Component | Observed  HR m/z | Theoretical High Res m/z |
| --- | --- | --- | --- |
| A (630-635) | PE(28:1) | 633.2681 | 633.5587253 |
|  |  |  |  |
| B (640-650) | PA(32:3) | 641.4188 | 641.4063305 |
|  | PA(32:2) | 643.4344 | 643.3889443 |
|  | PA(32:1) | 645.4501 | 645.4463521 |
|  | PG(a-28:2) | 647.4293 | 647.5755701 |
|  | PA(32:0) | 647.4657 | 647.5755701 |
|  | LPG(28:1) | 649.445 | 649.3981359 |
|  | PG(a-28:1) | 649.445 | 649.3981359 |
|  |  |  |  |
|  |  |  |  |
| C (650-660) | PE(P-32:0) | 659.2838 | 659.4666531 |
|  |  |  |  |
|  |  |  |  |
| D (675-680) | PA(34:2) | 671.4657 | 671.4648593 |
|  | PA(34:1) | 673.4814 | 673.4825289 |
|  | PA (32:3)+ Cl |  | 675.4 |
|  | PG(a-30:2 | 675.4606 | 675.5546883 |
|  | PA(34:0) | 675.497 | 675.5546883 |
|  | LPIP(18:1) | 677.2709 | 677.4325126 |
|  | LPI(24:3) | 677.3671 | 677.4325126 |
|  | PG(a-30:1) | 677.4763 | 677.4325126 |
|  | PA (32:2)+Cl |  | 677.7 |
|  | LPIP(18:0) | 679.2865 | 679.4501267 |
|  | LPI(24:2) | 679.3828 | 679.4501267 |
|  | PG(a-30:0) | 679.4919 | 679.4919282 |
|  |  |  |  |
|  |  |  |  |
| 690-695 | PE(32:0) | 690.5079 | 690.5813555 |
|  | PI(24:2) | 693.362 | 693.3969299 |
|  | PI(24:1) | 695.3777 | 695.513714 |
|  |  |  |  |
|  |  |  |  |
|  | PE(a-34:2) | 700.5286 | 700.5287331 |
| 700-705 | *PE 30:3 + Acetate* | 700.9 |  |
|  | LPIP(20:3) | 701.2709 | 701.5140139 |
|  | PG(a-32:3) | 701.4763 | 701.5140139 |
|  | PA(36:1) | 701.5127 | 701.5140139 |
|  | PE(a-34:1) | 702.5443 | 702.5121012 |
|  | LPIP(20:2) | 703.2865 | 703.5814769 |
|  | PG(a-32:2) | 703.4919 | 703.5814769 |
|  | PA(36:0) | 703.5283 | 703.5814769 |
|  |  |  |  |
|  |  |  |  |
| 709-720 | PG(32:4) | 713.4399 | 713.4566416 |
|  | PA(a-38:2) | 713.549 | 713.4910036 |
|  | PE(34:2) | 714.5079 | 714.5057608 |
|  | PG(32:3) | 715.4555 | 715.5059494 |
|  | PA(a-38:1) | 715.5647 | 715.5491615 |
|  | PE(34:1) | 716.5236 | 716.5225176 |
|  | PG(32:2) | 717.4712 | 717.526549 |
|  | PA(a-38:0) | 717.5803 | 717.526549 |
|  | PG(32:1) | 719.4868 | 719.4894421 |
|  |  |  |  |
|  |  |  |  |
| E (725-735) | PE(a-36:3) | 726.5443 | 726.5448417 |
|  | PG(a-34:4) | 727.4919 | 727.5111233 |
|  | PA(38:2) | 727.5283 | 727.5111233 |
|  | PE(a-36:2) | 728.5599 | 728.4476613 |
|  | PG(a-34:3) | 729.5076 | 729.563557 |
|  | PA(38:1) | 729.544 | 729.563557 |
|  | PE(a-36:1) | 730.5756 | 730.5671836 |
|  | LPIP(22:2) | 731.3178 | 731.4917523 |
|  | PG(a-34:2 | 731.5232 | 731.4917523 |
|  | PA(38:0) | 731.5596 | 731.568503 |
|  | PE(a-36:0) | 732.5912 | 732.5542077 |
|  | LPIP(22:1) | 733.3335 | 733.538853 |
|  | PI(a-28:3) | 733.4297 | 733.538853 |
|  | PG(a-34:0) | 735.5545 | 735.9806819 |
|  |  |  |  |
|  |  |  |  |
| F (740-750) | PE(36:3) | 740.5236 | 740.5236712 |
|  | PG(34:4) | 741.4712 | 741.5271404 |
|  | PA(a-40:2) | 741.5803 | 741.5641895 |
|  | PE(36:2) | 742.5392 | 742.5394215 |
|  | PG(34:3) | 743.4868 | 743.5012749 |
|  | PA(a-40:1) | 743.596 | 743.5823612 |
|  | PE(36:1) | 744.5549 | 744.5566235 |
|  | PE(36:0) | 746.5705 | 746.5547351 |
|  | PA(40:5) | 749.5127 | 749.4947323 |
|  | PG(34:0) | 749.5338 | 749.4947323 |
|  |  |  |  |
|  |  |  |  |
| G (750-760) | PE(P-38:4) | 750.5443 | 750.5006359 |
|  | PG(P-36:5) | 751.4919 | 751.5087064 |
|  | PA(40:4) | 751.5283 | 751.5087064 |
|  | PG(a-36:5) | 753.5076 | 753.5221125 |
|  | PA(40:3) | 753.544 | 753.5221125 |
|  | PG(a-36:4) | 755.5232 | 755.4965899 |
|  | PA(40:2) | 755.5596 | 755.4965899 |
|  | PG(a-36:3) | 757.5389 | 757.5576216 |
|  | PA(40:1) | 757.5753 | 757.5576216 |
|  | PA(40:0) | 759.5909 | 759.576202 |
|  |  |  |  |
| 760-765 | PG(a-36:1) | 761.5702 | 761.4951796 |
|  | PA(a-42:5) | 763.5647 | 763.5084482 |
|  | PG(a-36:0) | 763.5858 | 763.5084482 |
|  | PE(38:5) | 764.5236 | 764.5175277 |
|  | PA(a-42:4) | 765.5803 | 765.4164385 |
|  | PG(36:6) | 765.4712 | 765.5225897 |
|  |  |  |  |
| H (770-780) | PE(38:2) | 770.5705 | 770.5701211 |
|  | PG(36:3) | 771.5181 | 771.5177348 |
|  | PA(a-42:1), PA(P-42:0) | 771.6273 | 771.6266341 |
|  | PG(36:2) | 773.5338 | 773.5339366 |
|  | PA(a-42:0) | 773.6429 | 773.5914984 |
|  | PIP(24:1) | 775.344 | 775.508051 |
|  | PI(30:3) | 775.4403 | 775.508051 |
|  | PA(42:6) | 775.5283 | 775.508051 |
|  | PG(36:1) | 775.5494 | 775.5499889 |
|  | PIP(24:0) | 777.3597 | 777.525509 |
|  | PI(30:2) | 777.4559 | 777.525509 |
|  | PA(42:5) | 777.544 | 777.525509 |
|  | PG(36:0) | 777.5651 | 777.525509 |
|  | PI (30:1) |  | 779.5 |
|  |  |  |  |
|  |  |  |  |
| 780-790 | PI(30:0) | 781.4872 | 781.4899445 |
|  | PA(P-44:8) | 783.5334 | 783.5310831 |
|  | PG(a-38:4) | 783.5545 | 783.5310831 |
|  | PA(42:2) | 783.5909 | 783.5310831 |
|  | PA(a-44:7) | 787.5647 | 787.5702346 |
|  | PG(a-38:2) | 787.5858 | 787.5702346 |
|  | PA(42:0) | 787.6222 | 787.5702346 |
|  | PIP (P-26:0) |  | 789 |
|  |  |  |  |
|  |  |  |  |
| 790-800 | PE(40:6) | 790.5392 | 790.566426 |
|  | PE(40:5) | 792.5549 | 792.5316314 |
|  | PE(40:4) | 794.5705 | 794.5186555 |
|  | PE(40:3) | 796.5862 | 796.5457723 |
|  | PE(40:2) | 798.6018 | 798.5618072 |
|  |  |  |  |
|  |  |  |  |
| I (800-810) | PE(40:1) | 800.6175 | 800.5846771 |
|  | PE(P-42:7) | 800.5599 | 800.5846771 |
|  | PS(a-38:2) | 800.5811 | 800.5846771 |
|  | PE(40:0) | 802.6331 | 802.5703329 |
|  | PS(a-38:1) | 802.5967 | 802.5703329 |
|  | PS(a-38:0) | 804.6124 | 804.5772529 |
|  | PI(32:1) | 807.5029 | 807.5036969 |
|  | PI(32:0) | 809.5185 | 809.5100099 |
|  | PG(a-40:5) | 809.5702 | 809.5651801 |
|  | PA(44:3) | 809.6066 | 809.5651801 |
|  |  |  |  |
|  |  |  |  |
| J (815-825) | PI(a-34:4) | 815.508 | 815.540458 |
|  | PE(42:5) | 820.5862 | 820.5626888 |
|  | PG(40:6) | 821.5338 | 821.5162019 |
|  | PI(a-34:1) | 821.5549 | 821.5677079 |
|  | PA(a-46:4) | 821.6429 | 821.642413 |
|  | PE(42:4) | 822.6018 | 822.5845851 |
|  | PI(a-34:0) | 823.5706 | 823.5621416 |
|  | PG(40:5) | 823.5494 | 823.5621416 |
|  | PA(a-46:3) | 823.6586 | 823.59022 |
|  | PE(42:3) | 824.6175 | 824.5734332 |
|  | PIP(28:4) | 825.3597 | 825.5829589 |
|  | PI(34:6) | 825.4559 | 825.5829589 |
|  | PG(40:4) | 825.5651 | 825.5829589 |
|  | PA(a-46:2) | 825.6742 | 825.5829589 |
|  |  |  |  |
|  |  |  |  |
| K (830-840) | PE(42:0) | 830.6644 | 830.5909496 |
|  | PS(a-40:1) | 830.628 | 830.5909496 |
|  | PIP(28:1) | 831.4066 | 831.5920895 |
|  | PI(34:3) | 831.5029 | 831.5920895 |
|  | PA(46:6) | 831.5909 | 831.5920895 |
|  | PG(40:1) | 831.612 | 831.5920895 |
|  | PE(a-44:6) | 832.6225 | 832.597884 |
|  | PS(a-40:0) | 832.6437 | 832.597884 |
|  | PIP(28:0) | 833.4223 | 833.5193396 |
|  | PI(34:2) | 833.5185 | 833.5193396 |
|  | PG(40:0) | 833.6277 | 833.5538803 |
|  | PS(40:6) | 834.529 | 834.5255498 |
|  | PE(a-44:5) | 834.6382 | 834.5584476 |
|  | PI(34:1) | 835.5342 | 835.5349089 |
|  | PG(a-42:6) | 835.5858 | 835.5349089 |
|  | PA(46:4) | 835.6222 | 835.6529621 |
|  | PS(40:5) | 836.5447 | 836.5367826 |
|  | PE(a-44:4) | 836.6538 | 836.6018618 |
|  | PIP(P-30:4) | 837.3961 | 837.5416088 |
|  | PI(34:0) | 837.5498 | 837.5416088 |
|  | PG(a-42:5) | 837.6015 | 837.6075144 |
|  | PA(46:3) | 837.6379 | 837.6075144 |
|  | PE(a-44:3) | 838.6695 | 838.6106687 |
|  | PE(a-44:2) | 840.6851 | 840.5502944 |
|  | PS(40:3) | 840.576 | 840.5502944 |
|  |  |  |  |
|  |  |  |  |
| 840-845 | PIP(a-30:3) | 841.4274 | 841.4198356 |
|  | PI(a-36:5) | 841.5236 | 841.4998244 |
|  | PG(a-42:3) | 841.6328 | 841.4998244 |
|  | PA(46:1) | 841.6692 | 841.4998244 |
|  | PS(40:2) | 842.5916 | 842.5458527 |
|  | PIP(a-30:2) | 843.443 | 843.413187 |
|  | PI(a-36:4) | 843.5393 | 843.413187 |
|  |  |  |  |
|  |  |  |  |
| L (845-855) | PA(a-48:4) | 849.6742 | 849.4747784 |
|  | PI(a-36:1) | 849.5862 | 849.54976 |
|  | PG(42:6) | 849.5651 | 849.54976 |
|  | PS(a-42:5) | 850.5967 | 850.6200373 |
|  | PIP(30:5) | 851.3753 | 851.5553462 |
|  | PG(42:5) | 851.5807 | 851.5553462 |
|  | PI(a-36:0) | 851.6019 | 851.6215287 |
|  | PA(a-48:3) | 851.6899 | 851.6215287 |
|  | PS(a-42:4) | 852.6124 | 852.6244578 |
|  | PIP(30:4) | 853.391 | 853.5587146 |
|  | PI(36:6) | 853.4872 | 853.5587146 |
|  | PA(a-48:2) | 853.7055 | 853.5587146 |
|  | PG(42:4) | 853.5964 | 853.6253363 |
|  |  |  |  |
| M (859-870) | PI(36:3) | 859.5342 | 859.534851 |
|  | PIP(30:1) | 859.4379 | 859.5365897 |
|  | PG(42:1) | 859.6433 | 859.5365897 |
|  | PS(a-42:0) | 860.675 | 860.5363813 |
|  | PI(36:2) | 861.5498 | 861.5500901 |
|  | PIP(30:0) | 861.4536 | 861.551467 |
|  | PA(48:5) | 861.6379 | 861.6458106 |
|  | PG(42:0) | 861.659 | 861.6458106 |
|  | PI(36:1), IPC (20:0) | 863.5655 | 863.5661142 |
|  | PG(a-44:6) | 863.6171 | 863.6429703 |
|  | PA(48:4) | 863.6535 | 863.6429703 |
|  | PS(42:5) | 864.576 | 864.570496 |
|  | PI(36:0) | 865.5811 | 865.57085 |
|  | PG(a-44:5) | 865.6328 | 865.6400658 |
|  | PA(48:3) | 865.6692 | 865.6400658 |
|  | PS(42:4) | 866.5916 | 866.5747315 |
|  | PI(a-38:6 | 867.5393 | 867.6151897 |
|  | PG(a-44:4) | 867.6484 | 867.6151897 |
|  | PA(48:2) | 867.6848 | 868.5926975 |
|  | PS(42:3) | 868.6073 | 869.5934273 |
|  | PG(a-44:3) | 869.6641 | 869.5934273 |
|  | PA(48:1) | 869.7005 | 869.5934273 |
|  |  |  |  |
|  |  |  |  |
| N (875-885) | PI(a-38:2) | 875.6019 | 875.5654644 |
|  | PIP(a-32:0) | 875.5056 | 875.5654644 |
|  | IPC (22:0) | 877.4872 | 877.5816875 |
|  | PI(a-38:1) | 877.6175 | 879.5600897 |
|  | PIP(32:5) | 879.4066 | 879.5600897 |
|  | PI(a-38:0) | 879.6332 | 880.5649611 |
|  | PE(46:3) | 880.6801 | 881.5936523 |
|  | PIP(32:4) | 881.4223 | 881.5936523 |
|  | PG(44:4) | 881.6277 | 882.6091472 |
|  | PE(46:2) | 882.6957 | 883.5611649 |
|  | PIP(32:3) | 883.4379 | 883.5611649 |
|  | PI(38:5) | 883.5342 | 884.6138136 |
|  | PE(46:1) | 884.7114 | 885.4453592 |
|  | PIP(32:2) | 885.4536 | 885.6176746 |
|  | PI(38:4) | 885.5498 | 885.6176746 |
|  | PG(44:2) | 885.659 | 885.6176746 |
|  |  |  |  |
|  |  |  |  |
| O (905-910) | PI(40:8) | 905.5185 | 905.6480742 |
|  | PI(a-40:1) | 905.6488 | 905.6485378 |
|  | PIP(34:5) | 907.4379 | 907.5199892 |
|  | PI(40:7) | 907.5342 | 907.5199892 |
|  | PI(a-40:0) | 907.6645 | 907.6528258 |
|  |  |  |  |
| 920-925 | PI(40:1) | 919.6281 | 919.6665221 |
|  | PI(40:0) | 921.6437 | 921.6723635 |
|  |  |  |  |
| P (930-940) | PIP(a-36:0) | 931.5682 | 931.6640138 |
|  | PI(a-42:2) | 931.6645 | 931.6640138 |
|  | PIP(36:6) | 933.4536 | 933.6801706 |
|  | PI(42:8) | 933.5498 | 933.6801706 |
|  | PI(a-42:1) | 933.6801 | 933.6801706 |
|  | PIP(36:5) | 935.4692 | 935.6860051 |
|  | PI(42:7) | 935.5655 | 935.6860051 |
|  | PI(a-42:0) | 935.6958 | 935.6860051 |

Table S3 Enzymes involved in *C. fasiculata* inositol lipid metabolism. Gene information obtained from Tri-tryp gene database*.* * denotes extra *Crithidia* homologs present

| Biosynthetic Step | Enzyme name | *C. fasiculata* gene | *L. mexicana* gene | *T. brucei* gene |
| --- | --- | --- | --- | --- |
| INO1 | Inositol-3-phosphate synthase | CfaC1_11_1650 | LmxM.14.1360 | Tb927.10.7110 |
| Table 3 IMPase 1 | Inositol Monophosphatase 1 | CFAC1_240041200 | LmxM.15.0880 | Tb927.9.6350 |
| IMPase 2 | Inositol Monophosphatase 2 | CFAC1_090022500 | LmxM.17.1390 | Tb927.5.2690 |
| PIK | PI3 kinase Class (III) | CfaC1_30_4130 | LmxM.24.2010 | Tb927.8.6210 |
| PIK | PI4 kinase | CfaC1_33_5050 | LmxM.33.3590 | Tb927.4.1140 |
| PIK | PI4 kinase α | CfaC1_25_1690 | LmxM.08_29.1450 | Tb927.3.4020 |
| PIK | PIK-related | CfaC1_23_1350 | LmxM.20.1120 | Tb927.1.1930 |
| PIK | PIK-related | CfaC1_32_1950* | - | - |
| PIS | Phosphatidylinositol synthase | CfaC1_33_0340 | LmxM.26.2480 | Tb927.9.1610 |
| SLS | IPC synthase | CfaC1_31_5160 | LmxM.34.4990 | Tb927.9.9380 |

Table S4 – Inositol containing lipids identified in *C. fasiculata* total lipid extracts from high resolution ESI-MS data. Species assignments are based on MS-MS daughter fragment data collected as detailed in experimental methods. Species highlighted with * indicates an observed mass within 0.02 Da of theoretical.

| Peak | m/z | Lipid Component | Principle Component | Theoretical m/z | HR m/z |
| --- | --- | --- | --- | --- | --- |
| A (#%) |  |  |  |  |  |
|  | 625.32 | LPI(20:1) |  | 625.3358* | 625.3211 |
|  | 627.35 | LPI(20:0) |  | 627.3515* | 627.3526 |
|  |  |  |  |  |  |
| B (#%) |  |  |  |  |  |
|  | 683.42 | LPI(24:0) |  | 683.4141* | 683.4155 |
|  |  |  |  |  |  |
| C (#%) |  |  |  |  |  |
|  | 699.29 | LPIP(20:4) |  | 699.2552 | 699.2915 |
|  |  |  |  |  |  |
| D (#%) |  |  |  |  |  |
|  | 735.40 | LPIP(22:0) |  | 735.3491 | 735.3989 |
|  | 735.48 | PI(a-28:2) |  | 735.4454 | 735.4793 |
| E (#%) |  |  |  |  |  |
|  | 778.52 | IPC 34:1 |  | 778.5240* | 778.5185 |
|  | 779.50 | PI (30:1) | PI 12:0/18:1 or PI 14:0/16:1 | 779.4716 | 779.583 |
|  | 780.53 | IPC 34:0 |  | 780.5396* | 780.532 |
|  | 781.52 | PI(30:0) |  | 781.4872 | 781.522 |
|  | 789.00 | PIP (a-26:1) | PIP (a-16:1/10:0) | 709.4297* | 709.4266 |
| F (#%) |  |  |  |  |  |
|  | 804.53 | IPC 36:1 |  | 804.5397* | 804.5272 |
|  | 806.55 | IPC 36:0 |  | 806.5553* | 806.5481 |
|  | 807.50 | PI(32:1) | PI 14:0/18:1 | 807.5029* | 807.5034 |
|  | 809.51 | PI(32:0) |  | 809.5185* | 809.5095 |
|  | 813.48 | PI(a-34:5) |  | 813.4923* | 813.4799 |
|  |  |  |  |  |  |
| G (#%) |  |  |  |  |  |
|  | 821.52 | PI(a-34:1) | PI a-16:0/18:1 | 821.5549* | 821.5159 |
|  | 823.56 | PI(a-34:0) | PI a-16:0/18:0 | 823.5706* | 823.5635 |
|  | 825.50 | PI(34:6) |  | 825.4559 | 825.4955 |
|  | 827.50 | PI(34:5) |  | 827.4716 | 827.4954 |
|  | 829.48 | PI(34:4) |  | 829.4872* | 829.4847 |
|  |  |  |  |  |  |
| H (#%) |  |  |  |  |  |
|  | 831.41 | PIP(28:1) |  | 831.4066* | 831.4103 |
|  | 831.50 | PI(34:3) |  | 831.5029* | 831.5009 |
|  | 833.52 | PI(34:2) |  | 833.5185* | 833.52 |
|  | 833.56 | PI(a-36:1) |  | 833.5913 | 833.5552 |
|  | 834.56 | IPC 38:1 |  | 834.5866 | 834.5575 |
|  | 835.53 | PI(34:1) |  | 835.5342* | 835.5348 |
|  | 836.60 | IPC 38:0 |  | 836.6022* | 836.6019 |
|  | 837.54 | PI(34:0) | PI 16:0/18:0 | 837.5498* | 837.5407 |
|  | 839.55 | PI(a-36:6) |  | 839.508 | 839.5457 |
|  | 841.51 | PI(a-36:5) |  | 841.5236* | 841.5061 |
| I (#%) |  |  |  |  |  |
|  | 849.46 | PI(36:8) |  | 849.4559* | 849.4617 |
|  | 849.55 | PI(a-36:1) | PI a-18:0/18:1 | 849.5862* | 849.5502 |
|  | 851.47 | PI(36:7) |  | 851.4716* | 851.4749 |
|  | 851.62 | PI(a-36:0) |  | 851.6019* | 851.6216 |
|  | 855.51 | PI(36:5) |  | 855.5029* | 855.5057 |
|  | 857.52 | PI(36:4) |  | 857.5185* | 857.5194 |
|  | 859.45 | PIP(30:1) |  | 859.4379* | 859.4536 |
|  | 859.53 | PI(36:3) |  | 859.5342* | 859.5347 |
| J (#%) |  |  |  |  |  |
|  | 861.44 | PIP(30:0) |  | 861.4536* | 861.4418 |
|  | 861.55 | PI(36:2) |  | 861.5498* | 861.5502 |
|  | 863.57 | PI(36:1) | PI 18:0/18:1 | 863.5655* | 863.5654 |
|  | 865.50 | PI(a-38:7) |  | 865.5236 | 865.5013 |
|  | 865.57 | PI(36:0) | PI 18:0/18:0 | 865.5811* | 865.5718 |
|  | 867.58 | PI(a-38:6) | PI a-16:0/22:5 | 867.5393 | 867.5816 |
|  | 869.60 | PI(a-38:5) |  | 869.5549 | 869.5952 |
|  | 871.52 | PIP(a-32:1) |  | 871.4743 | 871.5157 |
|  | 871.56 | PI(a-38:4) |  | 871.5706* | 871.5646 |
|  | 873.59 | PI(a-38:3) |  | 873.5862* | 873.5874 |
| K (#%) |  |  |  |  |  |
|  |  |  |  |  |  |
|  | 877.58 | PI(38:8) | PI 16:1/22:7 | 877.4872 | 877.5816875 |
|  | 877.66 | PI(a-38:1) | PI O-18:1/20:0 | 877.6175 | 877.6589 |
|  | 881.56 | PI(38:6) |  | 881.5185 | 881.5645 |
|  | 883.52 | PI(38:5) | PI 18:1/20:4 | 883.5342* | 883.5205 |
|  |  |  |  |  |  |
| L (#%) |  |  |  |  |  |
|  | 885.45 | PIP(32:2) |  | 885.4536* | 885.447 |
|  | 885.55 | PI(38:4) | PI 18:0/20:4 | 885.5498* | 885.5514 |
|  | 887.49 | PIP(32:1) |  | 887.4692 | 887.4913 |
|  | 887.56 | PI(38:3) |  | 887.5655* | 887.5628 |
|  | 889.48 | PIP(32:0) |  | 889.4849* | 889.4788 |
|  | 889.58 | PI(38:2) |  | 889.5811* | 889.5827 |
|  |  |  |  |  |  |
| M (#%) |  |  |  |  |  |
|  | 905.53 | PI(40:8) |  | 905.5185* | 905.5274 |
|  | 905.65 | PI(a-40:1) |  | 905.6488* | 905.6488 |
|  | 907.52 | PI(40:7) |  | 907.5342* | 907.5192 |
|  | 907.65 | PI(a-40:0) |  | 907.6645* | 907.6545 |
|  | 909.53 | PI(40:6) |  | 909.5498* | 909.5322 |
|  | 911.54 | PI(40:5) | PI 18:0/22:5 | 911.5655 | 911.5424 |
|  |  |  |  |  |  |
| N (#%) |  |  |  |  |  |
|  | 919.50 | PIP(a-36:6) |  | 919.4743 | 919.503 |
|  | 919.66 | PI(40:1) | PI 16:1/24:0 | 919.6281 | 919.6645 |
|  | 921.52 | PIP(a-36:5) |  | 921.49 | 921.5208 |
|  | 921.67 | PI(40:0) |  | 921.6437 | 921.6704 |
|  | 923.51 | PIP(a-36:4) |  | 923.5056* | 923.5146 |
|  | 925.53 | PIP(a-36:3) |  | 925.5213* | 925.53 |
|  | 925.65 | PI(a-42:5) |  | 925.6175 | 925.6531 |
|  |  |  |  |  |  |
| O (#%) |  |  |  |  |  |
|  | 931.47 | PIP(36:7) |  | 931.4379 | 931.4696 |
|  | 931.66 | PI(a-42:2) |  | 931.6645* | 931.6646 |
|  | 933.53 | PI(42:8) |  | 933.5498* | 933.5314 |
|  | 935.51 | PIP(36:5) |  | 935.4692 | 935.5056 |
|  | 935.69 | PI(a-42:0) |  | 935.6958* | 935.6867 |
|  | 937.49 | PIP(36:4) |  | 937.4849* | 937.4928 |
|  | 937.55 | PI(42:6) |  | 937.5811 | 937.5454 |
|  | 939.51 | PIP(36:3) |  | 939.5005* | 939.5084 |
|  | 939.59 | PI(42:5) |  | 939.5968* | 939.5918 |

Table S5– Enzymes involved in *C. fasiculata* choline lipid metabolism. Gene information obtained from Tri-tryp gene database. * denotes extra *Crithidia* homologs present

| Biosynthetic Step | Enzyme name | *C. fasiculata* gene | *L. mexicana* gene | *T. brucei* gene |
| --- | --- | --- | --- | --- |
| CK | Choline kinase 2 | CfaC1_20_1660 | LmxM.27.1420 | Tb927.11.2090 |
| CCT | Choline-phosphate cytidylyltransferase | CfaC1_21_1850 | LmxM.18.1330 | Tb927.10.12810 |
| CPT | Choline phosphotransferase | CfaC1_13_0220 | LmxM.03.0821 | Tb927.10.8900 |
| CPT | Choline phosphotransferase | CfaC1_35_5820* | - | - |
| PEMT | Phosphatidylethanolamine N-methyltransferase (Class I) | CfaC1_34_4100 | LmjF.31.2290 | No Homologue |
| PEMT | Phosphatidylethanolamine N-methyltransferase (Class II) | CfaC1_34_5650 | LmjF.31.3120 | No Homologue |
| PLA1 | Phospholipase A1 | CfaC1_31_3310 | LmxM.34.3020 | Tb927.9.12700 |

Table S6 PC containing lipids identified in *C. fasiculata* total lipid extracts from high resolution ESI-MS data. Species assignments are based on MS-MS daughter fragment data collected as detailed in experimental methods. Species highlighted with a * denote an observed mass within 0.02 Da of theoretical

| Peak | m/z | Lipid Component | Principle Component | Theoretical m/z | HR m/z |
| --- | --- | --- | --- | --- | --- |
| A |  |  |  |  |  |
|  | 730.53 | PC(32:2) |  | 730.5382* | 730.5251 |
|  | 732.55 | PC(32:1) |  | 732.5538* | 732.5511 |
|  | 734.56 | PC(32:0) |  | 734.5695* | 734.5598 |
|  | 738.54 | PC(a-34:4) |  | 738.5432* | 738.541 |
|  | 740.55 | PC(a-34:4) |  | 740.5589* | 740.5524 |
|  |  |  |  |  |  |
| B |  |  |  |  |  |
|  | 746.57 | PC(a-34:1) |  | 746.6058 | 746.5691 |
|  | 748.58 | PC(a-34:0) |  | 748.6215 | 748.5765 |
|  | 750.48 | [PC(34:6)](http://www.lipidmaps.org/tools/ms/G_expand.php?ABBREV=PC%2834:6%29) |  | 750.5069 | 750.4817 |
|  | 752.56 | PC(34:5) |  | 752.5225 | 752.5564 |
|  | 754.54 | PC(34:4) |  | 754.5382* | 754.5366 |
|  | 756.54 | PC(34:3) |  | 756.5538* | 756.5416 |
|  |  |  |  |  |  |
| C |  |  |  |  |  |
|  | 758.57 | PC(34:2) |  | 758.5695* | 758.5697 |
|  | 760.59 | PC(34:1) |  | 760.5851* | 760.5852 |
|  | 762.59 | PC(34:0) | PC 16:0/18:0 | 762.6008* | 762.5922 |
|  | 764.51 | PC(a-36:6) |  | 764.5589 | 764.5109 |
|  | 768.55 | PC(a-36:4) |  | 768.5902 | 768.5513 |
|  | 770.56 | PC(a-36:3) |  | 770.6058 | 770.5572 |
|  |  |  |  |  |  |
| D |  |  |  |  |  |
|  | 772.59 | PC(a-36:2) |  | 772.6215 | 772.5853 |
|  | 774.47 | PC(36:8) |  | 774.5069 | 774.4662 |
|  | 774.60 | PC(a-36:1) |  | 774.6371 | 774.6007 |
|  | 776.48 | PC(36:7) |  | 776.5225 | 776.4846 |
|  | 776.61 | PC(a-36:0) |  | 776.6528 | 776.6084 |
|  | 778.51 | PC(36:6) | PC 14:0/22:6 | 778.5382 | 778.5123 |
|  | 780.55 | PC(36:5) |  | 780.5538* | 780.5511 |
|  | 782.57 | PC(36:4) |  | 782.5695* | 782.5683 |
|  |  |  |  |  |  |
| E |  |  |  |  |  |
|  | 784.59 | PC(36:3) |  | 784.5851* | 784.5856 |
|  | 786.60 | PC(36:2) | PC 18:1/18:1 | 786.6008* | 786.6008 |
|  | 788.61 | PC(36:1) |  | 788.6164* | 788.6112 |
|  | 790.54 | PC(a-38:7) |  | 790.5745 | 790.5381 |
|  | 790.62 | PC(36:0) | PC 14:0/22:0  & PC 16:0/20:0 | 790.6321* | 790.6243 |
|  | 792.60 | PC(a-38:6) |  | 792.5902* | 792.6049 |
|  | 794.57 | PC(a-38:5) |  | 794.6058 | 794.5676 |
|  | 796.58 | PC(a-38:4) |  | 796.6215 | 796.5822 |
|  | 798.59 | PC(a-38:3) |  | 798.6371 | 798.5886 |
|  | 800.68 | PC(a-38:2) |  | 800.6528 | 800.6821 |
|  |  |  |  |  |  |
| F |  |  |  |  |  |
|  | 802.53 | PC(38:8) |  | 802.5382* | 802.5293 |
|  | 802.64 | PC(a-38:1) |  | 802.6684 | 802.6373 |
|  | 804.55 | PC(38:7) |  | 804.5538* | 804.5512 |
|  | 804.68 | PC(a-38:0) |  | 804.6841* | 804.6839 |
|  |  |  |  |  |  |
| G |  |  |  |  |  |
|  | 830.57 | PC(40:8) |  | 830.5695* | 830.5695 |
|  | 832.58 | PC(40:7) |  | 832.5851* | 832.5791 |
|  | 834.60 | PC(40:6) |  | 834.6008* | 834.5989 |
|  | 836.62 | PC(40:5) | PC 18:0/22:5 | 836.6164* | 836.6171 |
|  | 838.63 | PC(40:4) |  | 838.6321* | 838.6301 |
|  |  |  |  |  |  |
| H |  |  |  |  |  |
|  | 850.70 | PC(a-42:5) |  | 850.6684 | 850.6991 |
|  | 852.55 | PC(42:11) |  | 852.5538* | 852.5509 |
|  | 852.71 | PC(a-42:4) |  | 852.6841 | 852.7133 |
|  |  |  |  |  |  |
| I |  |  |  |  |  |
|  | 876.71 | PC(a-44:6) |  | 876.6841 | 876.7097 |
|  | 878.59 | PC(44:12) |  | 878.5695* | 878.5891 |
|  | 878.73 | PC(a-44:5) |  | 878.6997 | 878.7277 |
|  | 880.74 | PC(a-44:4) |  | 880.7154 | 880.7447 |
|  |  |  |  |  |  |
| J |  |  |  |  |  |
|  | 909.78 | PC(a-44:1) + Na |  | 909.7515 | 909.7837 |
|  | 911.77 | PC(a-44:0) |  | 911.7670* | 911.7664 |
| K |  |  |  |  |  |
|  | 921.75 | PC(44:2) + Na |  | 921.7152 | 921.7511 |
|  | 923.77 | PC(44:1) + Na |  | 923.7308 | 923.7667 |
|  | 925.78 | [PC(44:0) + Na](http://www.lipidmaps.org/tools/ms/G_expand.php?ABBREV=PC%2846:2%29) |  | 925.7465 | 925.7772 |
| L |  |  |  |  |  |
|  | 939.63 | PC a-46:0 + Na |  | 939.7985 | 939.6331 |
|  | 943.61 | [PC(46:5)](http://www.lipidmaps.org/tools/ms/G_expand.php?ABBREV=PC%2846:5%29) + Na |  | 943.6995 | 943.6129 |

Table S7 Ethanolamine lipid metabolism. Gene information obtained from Tri-tryp gene database. * denotes extra *Crithidia* homologs present

| Biosynthetic Step | Enzyme name | *C. fasiculata* gene | *L. mexicana* gene | *T. brucei* gene |
| --- | --- | --- | --- | --- |
| EK | Ethanolamine kinase 1 | CfaC1_31_1320 | LmxM.34.1470 | Tb927.5.1140 |
| ECT | Ethanolamine-phosphate cytidyltransferase | CfaC1_32_1220 | LmxM.31.0890 | Tb927.11.14140 |
| EPT | Ethanolamine phosphotransferase | CfaC1_21_1090 | LmxM.18.0810 | Tb927.10.13290 |
| PEMT | Phosphatidylethanolamine N-methyltransferase | CfaC1_34_4100 | LmxM.30.2290 | NoHomologue |
| PEMT | Phosphatidylethanolamine N-methyltransferase | CfaC1_34_5650* |  | No Homologue |
| PSD | Phosphatidylserine decarboxylase | CfaC1_31_4780 | LmxM.34.4590 | Tb927.9.10080 |
| PSS/PSS2 | PS synthase/PS synthase-2 | CfaC1_11_1460 | LmxM.14.1200 | Tb927.7.3760 |
| SPL | Sphingosine-1-phosphate lyase | CfaC1_29_2570 | LmxM.29.2350 | Tb927.6.3630 |

Table S8 PE containing lipids identified in *C. fasiculata* total lipid extracts from high resolution ESI-MS data. Species assignments are based on MS-MS daughter fragment data collected as detailed in experimental methods. Species highlighted with * indicates an observed mass within 0.02 Da of theoretical.

| Masses | Species | Observed Mass | Theoretical Mass | Delta |
| --- | --- | --- | --- | --- |
| 714 | PE a-16:1/C19Δ | 714.5446 | 714.5443 | 0.0003 |
| 716 | PE a-16:0/C19Δ | 716.5514 | 716.5600 | 0.0086 |
| 728 | PE 16:1/C19Δ | 728.5159 | 728.5236 | 0.0077 |
| 730 | PE 16:0/C19Δ | 730.5668 | 730.5392 | 0.0276 |
| 742 | PE a-18:1/C19Δ | 742.576 | 742.5756 | 0.0004 |
| 744 | PE a-18:0/C19Δ | 744.5567 | 744.5913 | 0.0346 |
| *752* | PE 18:3/C19Δ | 752.5151 | 752.5236 | 0.0085 |
| 754 | PE 18:2/C19Δ | 754.5388 | 754.5392 | 0.0004 |
| 756 | PE 18:1/C19Δ | 756.5551 | 756.5549 | 0.0002 |


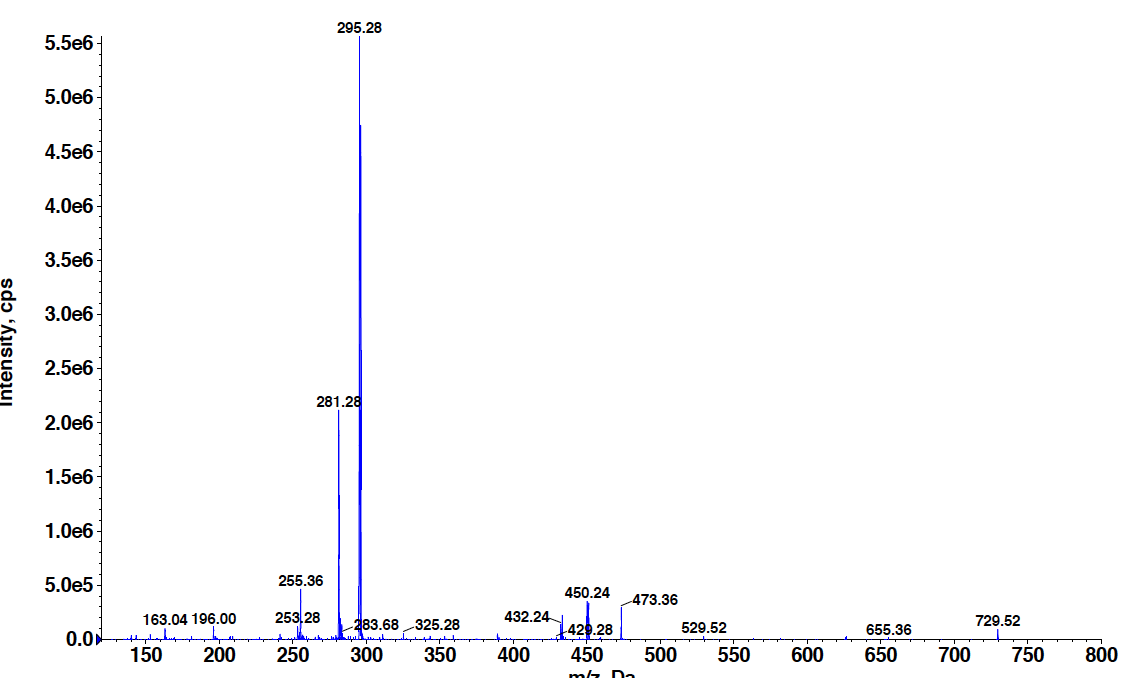


Figure S2 ESI-MS-MS daughter ion scanning in the negative ion mode of PE 35:0 (m/z 730) identified from *C. fasiculata* lipid. Ions used in assignment acyl composition.


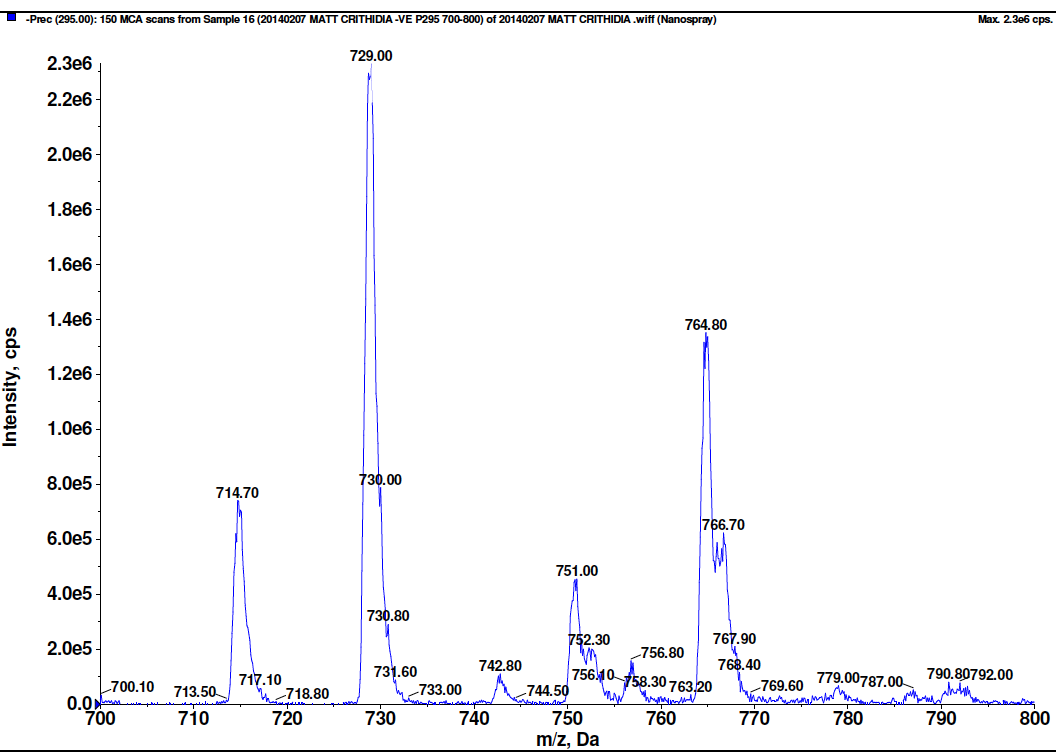


Figure S3: ESI-MS-MS negative parent ion scan (m/z 295) of *C. fasiculata* lipid extract showing C19Δ fatty acid containing species between m/z 700-800.

Table S9– Enzymes involved in *C. fasiculata* serine metabolism. Gene information obtained from Tri-tryp gene database. * denotes extra *Crithidia* homologs present

| Biosynthetic Step | Enzyme name | *C. fasiculata* gene | *L. mexicana* gene | *T. brucei* gene |
| --- | --- | --- | --- | --- |
| PSS2 | PS synthase-2 | CfaC1_11_1460 | LmxM.14.1200 | Tb927.7.3760 |
| PSD | Phosphatidylserine decarboxylase | CfaC1_31_4780 | LmxM.34.4590 | Tb927.9.10080 |
| SPT | Serine palmitoyltransferase | CfaC1_33_5220 | LmxM.33.3740 | Tb927.4.1020 |
| 3KSR | 3-Ketosphinganine reductase | CfaC1_24_2270 | LmxM.34.0330 | Tb927.10.4040 |
| DHCS | Dihydroceramide synthase | CfaC1_34_2800 | LmxM.30.1780 | Tb927.8.7730 |
| DHCD | Dihydroceramide desaturase | CfaC1_33_1220? | LmxM.26.1670 | Tb927.6.3000 |
| SK | Sphingosine kinase | CfaC1_08_0670 | LmxM.26.0710 | Tb927.7.1240 |
| SPL | Sphingosine-1-phosphate lyase | CfaC1_29_2570 | LmxM.29.2350 | Tb927.6.3630 |
| SPL | Sphingosine-1-phosphate lyase | CfaC1_29_2580* |  |  |
| SLS | Sphingolipid synthase 1-4 | CfaC1_31_5160 | LmxM.34.4990 | Tb927.9.9380 |
| nSMase/ISCL | Neutral sphingomyelinase/ Inositol phosphosphingolipid phospholipase C-Like protein | CfaC1_25_0400 | LmxM.08.0200 (LmjF.08.0200) | Tb927.5.3710 |

Table S10 PS lipids identified in *C. fasiculata* total lipid extracts from high resolution ESI-MS data. Species assignments are based on MS-MS daughter fragment data collected as detailed in experimental methods. Species highlighted with * indicates an observed mass within 0.02 Da of theoretical.

| M | m/z | Lipid Component | Principle Component | Theoretical m/z | HR m/z |
| --- | --- | --- | --- | --- | --- |
|  |  |  |  |  |  |
| A (#%) |  |  |  |  |  |
|  | 800.58 | [PS(a-38:2)](http://www.lipidmaps.org/tools/ms/G_expand.php?ABBREV=PS%28O-38:2%29) |  | 800.5811* | 800.5812 |
|  | 802.57 | [PS(a-38:1)](http://www.lipidmaps.org/tools/ms/G_expand.php?ABBREV=PS%28O-38:1%29) |  | 802.5967 | 802.5695 |
|  | 804.58 | [PS(a-38:0)](http://www.lipidmaps.org/tools/ms/G_expand.php?ABBREV=PS%28O-38:0%29) |  | 804.6124 | 804.5769 |
|  |  |  |  |  |  |
| B (#%) |  |  |  |  |  |
|  | 818.55 | [PS(a-40:7)](http://www.lipidmaps.org/tools/ms/G_expand.php?ABBREV=PS%28P-40:6%29) |  | 818.5341* | 818.5488 |
|  | 818.61 | [PS(38:0)](http://www.lipidmaps.org/tools/ms/G_expand.php?ABBREV=PS%2838:0%29) |  | 818.5916 | 818.6129 |
|  | 820.56 | [PS(a-40:6)](http://www.lipidmaps.org/tools/ms/G_expand.php?ABBREV=PS%28O-40:6%29) |  | 820.5498* | 820.5639 |
|  |  |  |  |  |  |
| C (#%) |  |  |  |  |  |
|  | 828.51 | [PS(40:9)](http://www.lipidmaps.org/tools/ms/G_expand.php?ABBREV=PS%2840:9%29) |  | 828.4821 | 828.5144 |
|  | 828.64 | [PS(a-40:2)](http://www.lipidmaps.org/tools/ms/G_expand.php?ABBREV=PS%28O-40:2%29) |  | 828.6124 | 828.6431 |
|  | 830.51 | [PS(40:8)](http://www.lipidmaps.org/tools/ms/G_expand.php?ABBREV=PS%2840:8%29) | PS 18:3/22:5 and PS 20:4/20:4 | 830.4977* | 830.5133 |
|  | 830.59 | [PS(a-40:1)](http://www.lipidmaps.org/tools/ms/G_expand.php?ABBREV=PS%28O-40:1%29) | PS a-20:0/20:1 | 830.628 | 830.5926 |
|  | 832.52 | PS(40:7) | PS 18:2/22:5 and PS 20:3/20:4 | 832.5134* | 832.5162 |
|  | 832.62 | PS(a-40:0) | PS a-20:0/20:0 | 832.6437* | 832.6248 |
|  | 834.53 | PS(40:6) | PS 18:1/22:5 | 834.529* | 834.526 |
|  |  |  |  |  |  |
| D (#%) |  |  |  |  |  |
|  | 840.61 | [PS(40:3)](http://www.lipidmaps.org/tools/ms/G_expand.php?ABBREV=PS%2840:3%29) |  | 840.576* | 840.613 |
|  | 842.59 | [PS(40:2)](http://www.lipidmaps.org/tools/ms/G_expand.php?ABBREV=PS%2840:2%29) |  | 842.5916* | 842.5927 |
|  | 844.56 | [PS(40:1)](http://www.lipidmaps.org/tools/ms/G_expand.php?ABBREV=PS%2840:1%29) |  | 844.6073 | 844.5647 |
|  |  |  |  |  |  |
| E (#%) |  |  |  |  |  |
|  | 854.50 | [PS(42:10)](http://www.lipidmaps.org/tools/ms/G_expand.php?ABBREV=PS%2842:10%29) |  | 854.4977* | 854.5009 |
|  | 854.63 | [PS(a-42:3)](http://www.lipidmaps.org/tools/ms/G_expand.php?ABBREV=PS%28O-42:3%29) |  | 854.628* | 854.6302 |
|  | 856.63 | [PS(a-42:2)](http://www.lipidmaps.org/tools/ms/G_expand.php?ABBREV=PS%28O-42:2%29) |  | 856.6437* | 856.6349 |
|  | 858.52 | [PS(42:8)](http://www.lipidmaps.org/tools/ms/G_expand.php?ABBREV=PS%2842:8%29) |  | 858.529* | 858.5226 |
|  | 858.62 | [PS(a-42:1)](http://www.lipidmaps.org/tools/ms/G_expand.php?ABBREV=PS%28O-42:1%29) |  | 858.6593 | 858.6208 |
|  | 860.54 | [PS(42:7)](http://www.lipidmaps.org/tools/ms/G_expand.php?ABBREV=PS%2842:7%29) |  | 860.5447* | 860.538 |
|  | 862.55 | [PS(42:6)](http://www.lipidmaps.org/tools/ms/G_expand.php?ABBREV=PS%2842:6%29) |  | 862.5603* | 862.5536 |
|  |  |  |  |  |  |
| F (#%) |  |  |  |  |  |
|  | 864.57 | [PS(42:5)](http://www.lipidmaps.org/tools/ms/G_expand.php?ABBREV=PS%2842:5%29) |  | 864.576* | 864.5691 |
|  | 866.57 | [PS(42:4)](http://www.lipidmaps.org/tools/ms/G_expand.php?ABBREV=PS%2842:4%29) |  | 866.5916* | 866.5747 |
|  | 868.59 | [PS(42:3)](http://www.lipidmaps.org/tools/ms/G_expand.php?ABBREV=PS%2842:3%29) |  | 868.6073* | 868.5921 |

Table S11 Enzymes involved in *C. fasiculata* fatty acid metabolism. Gene information obtained from Tri-tryp gene database. * denotes extra *Crithidia* homologs present

| Biosynthetic Step | Enzyme name | *C. fasiculata* gene | *L. mexicana* gene | *T. brucei* gene |
| --- | --- | --- | --- | --- |
| ACBP | Acyl-CoA binding protein | CFAC1_050015500 | LmxM.09.0750 | Tb427tmp.52.0001 |
| ACBP | Acyl-CoA binding protein | CFAC1_090012500 |  | Tb927.11.12830 |
| ACBP | Acyl-CoA binding protein | CFAC1_090012600* |  | Tb927.4.2010 |
| ACBP | Acyl-CoA binding protein | CFAC1_090012800* |  |  |
| ACBP | Acyl-CoA binding protein | CFAC1_280075400* |  |  |
| ACBP | Acyl-CoA binding protein | CFAC1_290055500* |  |  |
| ACC | Acetyl-CoA carboxylase | CfaC1_34_5430 | LmxM.30.2970 | Tb927.8.7100 |
| ACP | Acyl carrier protein | CfaC1_20_0290 | LmxM.27.0290 | Tb927.3.860 |
| ACS | Acyl-CoA synthetase | CfaC1_03_0320 | LmxM.03.0230 | Tb927.9.4190  (ACS1) |
| ACS | Acyl-CoA synthetase | CfaC1_12_0590 |  | Tb927.9.4200  (ACS2) |
| ACS | Acyl-CoA synthetase | CfaC1_12_0600 |  | Tb927.9.4210  (ACS3) |
| ACS | Acyl-CoA synthetase | CfaC1_12_0650 |  | Tb927.9.4230  (ACS4) |
| ACS | Acyl-CoA synthetase | CfaC1_12_0670 |  | Tb927.10.3260  (ACS5) |
| ACS | Acyl-CoA synthetase | CfaC1_12_0680 |  | Tb11.v5.0561 |
| ACS | Acyl-CoA synthetase | CfaC1_12_0690 | LmxM.01.0500 | Tb11.v5.0825 |
| ACS | Acyl-CoA synthetase | CfaC1_12_0700* |  |  |
| ACS | Acyl-CoA synthetase | CfaC1_12_0720* |  |  |
| ACS | Acyl-CoA synthetase | CfaC1_12_0730* |  |  |
| CFAS | Cyclopropyl Fatty acyl phospholipid Synthase | CfaC1_25_0770 | LmxM.08.0545 | No Homologue |
| MAT | acyl transferase-like protein | CFAC1_300085100 | LmxM.34.3470 | Tb927.9.11900 |
| ELO 1-4 | Fatty Acid Elongase | CfaC1_11_0820 | LmxM.14.0680 | Tb927.7.4160  (ELO1) |
| ELO 1-4 | Fatty Acid Elongase | CFAC1_110015600 | LmxM.14.0670 | Tb927.7.4170  (ELO2) |
| ELO 1-4 | Fatty Acid Elongase | CfaC1_11_0740 | LmxM.14.0640 | Tb927.7.4180  (ELO3) |
| ELO 1-4 | Fatty Acid Elongase | CfaC1_11_0790 | LmxM.14.0705 | Tb927.5.4530  (ELO4) |
| ELO 1-4 | Fatty Acid Elongase | CfaC1_11_0910 | LmxM.14.0740 |  |
| ELO 1-4 | Fatty Acid Elongase | CfaC1_32_0600* |  |  |
| ELO 1-4 | Fatty Acid Elongase | CfaC1_05_1300 | LmxM.05.1170 | Tb927.5.4530 |
| ELO 1-4 | Fatty Acid Elongase | CfaC1_18_1170 | LmxM.31.1160 | - |
| FAS II | β-ketoacyl-ACP reductase | CfaC1_30_3910 | LmxM.24.1810 | Tb927.8.6420 |
| FAS II | Enoyl-ACP reductase | CfaC1_27_1990 | LmxM.27.2440 | Tb927.2.5210 |
| FAS II | Enoyl-ACP reductase | CfaC1_27_1760* |  |  |
| FAS II | Enoyl-ACP reductase | CfaC1_30_4150* |  |  |
| FAS II | Enoyl-ACP reductase | CfaC1_33_2110* |  |  |
| FAS II | β-ketoacyl-ACP synthase | CfaC1_27_2870 | LmxM.32.2720 | Tb927.2.3910 |
| FAS II | β-ketoacyl-ACP dehydratase | CFAC1_080011300 | LmjF.07.0440 | No Homologue |
| FAS II | β-ketoacyl-ACP dehydratase | CFAC1_080011400 | LmjF.07.0430 | No Homologue |
| GAT 1 | Glycerol-3-phosphate acyltransferase | CfaC1_03_0170 | LmxM.03.0080 | Tb927.10.3100 |
| GAT 1 | Glycerol-3-phosphate acyltransferase | CfaC1_32_2520 | LmxM.31.1960 | Tb927.11.15150 |
| GAT 2 | 1-acyl-sn-glycerol-3-phosphateacyltransferase | CFAC1_190031500 | LmxM.31.1960 | Tb927.11.15150 |
| DAT | Dihydroxyacetonephosphate acyltransferase | CfaC1_33_2710 | LmxM.33.1090 | Tb927.4.3160 |
| ADS | 1-Alkyl-dihydroxyacetonephosphate synthase | CfaC1_29_0130 | LmxM.29.0120 | Tb927.6.1500 |
|  | CDP-DAG Synthase | CFAC1_290026600 | LmxM.26.1620 | Tb427.07.220 |
| PAP | Phosphatidic acid phosphatase | CfaC1_29_0470 | LmxM.29.0405 | Tb927.6.1820 |
| PAP | Phosphatidic acid phosphatase | CFAC1_140010800 | LmxM.19.1351 | Tb927.10.13930 |
| PAP | Phosphatidic acid phosphatase | CFAC1_150016500 | LmxM.23.1665 |  |
| DAGK | DAG kinase | CfaC1_17_1610 | LmxM.16.1290 | Tb927.8.5140 |
| DAGAT | DAG acyltransferase | CfaC1_20_1790 | LmxM.27.1560 | Tb927.11.2210 |
| DAGAT | DAG acyltransferase | CfaC1_20_1800* |  |  |
| DAGAT | DAG acyltransferase | CfaC1_20_1810* |  |  |
| DAGAT | DAG acyltransferase | CfaC1_02_0190* |  |  |
| PGPS | Phosphatidylglycerophosphate synthase | CfaC1_09_0360 | LmxM.07.0200 | Tb927.8.1720 |
| CLS | Cardolipin synthase | CfaC1_33_3450 | LmxM.33.2110 | Tb927.4.2560 |

Table S12. Fatty acid content in *C. fasciculata* WT cultured in standard fat (serum)-free media at 27°C and at 20°C. The table shows a summary of the relative abundance and the retention times of the FAMEs or FAs after GC-MS analysis of samples obtained from *C. fasciculata* WT grown in standard fat (serum)-free media at 27°C and at 20°C for 72 h. Values are the mean of three independent biological replicates (n=3). SD is standard deviation of each mean (±).

|  |  | *C. fasciculata* WT 27°C | | *C. fasciculata* WT 20°C | |
| --- | --- | --- | --- | --- | --- |
| FAME | Time (min) | Mean Relative Abundance (%) | SD | Mean Relative Abundance (%) | SD |
| 14:0 | 31.67 | 0.85 | 0.0241 | 1.48 | 0.0990 |
| 15:0 | 33.02 | 1.45 | 0.4251 | 4.11 | 0.0355 |
| 16:1 | 34.91 | 0.93 | 0.0071 | 0.83 | 0.0159 |
| 16:1 | 35.37 | 0.16 | 0.0137 | 1.16 | 0.0085 |
| 16:0 | 35.81 | 3.10 | 0.2999 | 1.93 | 0.0816 |
| Hydroxy-16:0 | 36.72 | 0.27 | 0.0250 | 0.24 | 0.0534 |
| Methyl-16:0 | 37.00 | 1.69 | 0.2018 | 1.18 | 0.0370 |
| 17:0 | 37.26 | 1.13 | 0.0743 | 0.92 | 0.0411 |
| 17:0Δ | 37.72 | 1.42 | 0.2951 | 1.84 | 0.0509 |
| 18:3 | 38.81 | 14.51 | 0.6638 | 14.87 | 0.7612 |
| 18:2 | 38.86 | 1.30 | 0.0900 | 4.03 | 0.0369 |
| 18:2 | 39.02 | 7.03 | 0.2333 | 2.81 | 0.3880 |
| 18:1 | 39.34 | 20.92 | 0.3604 | 24.62 | 1.3029 |
| 18:1 | 39.35 | 3.44 | 0.0430 | 1.72 | 0.0117 |
| 18:0 | 39.65 | 8.11 | 0.4210 | 8.80 | 0.1438 |
| 19:1Δ | 39.92 | 0.28 | 0.0428 | 3.47 | 0.0401 |
| 19:0Δ | 40.07 | 0.77 | 0.0178 | 2.68 | 0.1112 |
| Epoxy-18:0 | 40.66 | 0.90 | 0.0226 | 1.26 | 0.1144 |
| 19:0Δ | 41.10 | 8.44 | 0.1943 | 7.74 | 0.4688 |
| 20:4 | 41.91 | 0.56 | 0.0917 | 0.60 | 0.0754 |
| 20:4 | 42.21 | 0.91 | 0.0462 | 0.43 | 0.0964 |
| 20:3 | 42.30 | 0.10 | 0.0212 | 0.13 | 0.0291 |
| 20:3 | 42.49 | 0.15 | 0.0167 | 0.08 | 0.0091 |
| 20:2 | 42.59 | 0.06 | 0.0017 | 0.18 | 0.0124 |
| 20:2 | 42.98 | 0.16 | 0.0083 | 0.08 | 0.0602 |
| 20:1 | 43.42 | 0.22 | 0.0111 | 0.14 | 0.0100 |
| 20:1 | 43.45 | 0.16 | 0.0292 | 0.18 | 0.0033 |
| 20:0 | 43.96 | 0.35 | 0.0149 | 0.15 | 0.0221 |
| 22:5 | 45.09 | 14.59 | 0.1490 | 10.17 | 0.7970 |
| 22:4 | 45.23 | 0.90 | 0.0336 | 0.27 | 0.0357 |
| 22:4 | 46.01 | 0.23 | 0.0282 | 0.20 | 0.0048 |
| 22:3 | 46.18 | 0.08 | 0.0033 | 0.04 | 0.0033 |
| 22:3 | 46.41 | 0.24 | 0.0165 | 0.04 | 0.0059 |
| 22:2 | 46.52 | 0.37 | 0.0193 | 0.08 | 0.0016 |
| 22:2 | 47.01 | 0.10 | 0.0036 | 0.25 | 0.0247 |
| 22:0 | 47.16 | 0.21 | 0.0126 | 0.07 | 0.0109 |
| 24:0 | 47.72 | 0.66 | 0.0202 | 0.55 | 0.0195 |


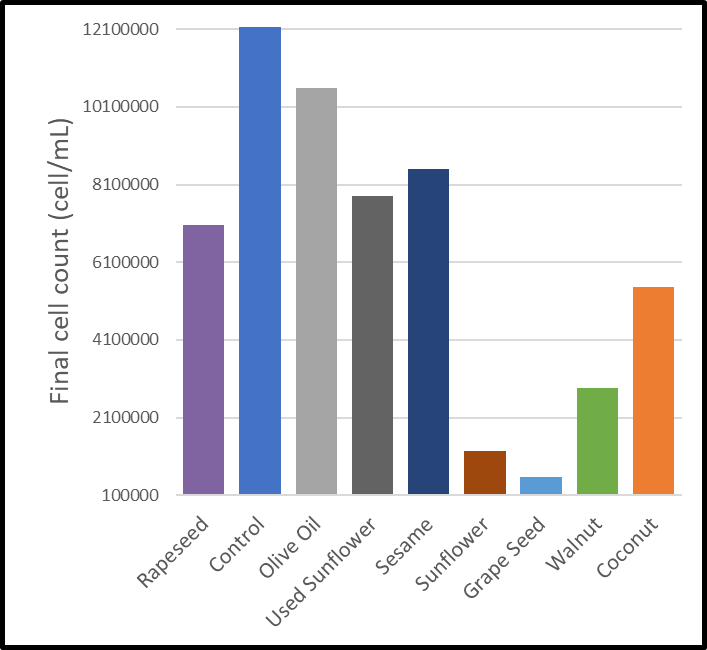


Figure S4. Cell density of *C. fasciculata* WT cultured upon supplementation with commercial oils. The bar chart shows the final cell density (Y axes) at the end of the growth curve performed for *C. fasciculata* WT cultured in media supplemented with various oil sources (X axis) at 27˚C for 48 h.

**A**

**B**

**C**

**D**

**E**

**F**

**G**

**H**

Figure S5. Comparison of fatty acids produced by *C. fasciculata* after supplementation with cooking oils.  The bar chart aims to give a visual summary of the of variation of FAs (Y axis) comparing the total of each class of FAs (X axis, part of total obtained by contingency table produced with PRISM 6.0) in *C. fasciculata* cultured in minimal media with 10% of Tween-80, and *C. fasciculata* grown in minimal media supplemented with 150 μL of sesame oil, rapeseed oil, walnut oil, olive oil, grapeseed oil, coconut oil, sunflower oil and used sunflower oil.

Table S13. Fatty acid content in C. fasciculata WT cultured in media supplemented with coconut oil at 27°C. The table shows a summary of the relative abundance and the retention times of the FAMEs or FAs after GC-MS analysis of samples obtained from the condition and naïve media supplemented with coconut oil, and of C. fasciculata WT control grown at 27°C for 48 h. Values are the mean of three independent biological replicates (n=3). SD is standard deviation of each mean (±).

|  |  | WT Control 27°C | | WT Coconut Oil 27°C | | Coconut Condition Media | | Naive Media | |
| --- | --- | --- | --- | --- | --- | --- | --- | --- | --- |
| FAME | Time (min) | Mean Relative Abundance (%) | SD | Mean Relative Abundance (%) | SD | Mean Relative Abundance (%) | SD | Mean Relative Abundance (%) | SD |
| 12:0 | 30.54 | 2.88 | 0.0657 | 5.67 | 0.0612 | 15.63 | 0.2271 | 16.96 | 0.0449 |
| 14:0 | 31.67 | 5.80 | 0.0896 | 12.15 | 0.0447 | 8.37 | 0.0501 | 12.46 | 0.0309 |
| 15:0 | 33.02 | 2.60 | 0.0927 | 1.19 | 0.0348 | NA | NA | NA | NA |
| 16:2 | 34.91 | 1.13 | 0.0633 | 0.54 | 0.0404 | NA | NA | NA | NA |
| 16:1 | 35.37 | 0.95 | 0.0189 | 1.60 | 0.0353 | NA | NA | NA | NA |
| 16:0 | 35.81 | 6.74 | 0.1102 | 11.55 | 0.1298 | 0.85 | 0.0380 | 8.48 | 0.0549 |
| 17:0 | 37.26 | NA | NA | NA | NA | 7.89 | 0.0433 | NA | NA |
| 17:0Δ | 37.72 | 2.87 | 0.0471 | 1.55 | 0.0726 | NA | NA | NA | NA |
| 18:3 | 38.81 | 3.25 | 0.0650 | 3.45 | 0.0601 | NA | NA | NA | NA |
| 18:2 | 38.86 | 4.26 | 0.0569 | 13.94 | 0.0757 | 0.40 | 0.0318 | 1.60 | 0.0162 |
| 18:1 | 39.34 | 51.26 | 0.1411 | 29.08 | 0.0433 | 51.50 | 0.2594 | 51.75 | 0.0545 |
| 18:1 | 39.35 | NA | NA | NA |  | 7.77 | 0.0498 | 6.05 | 0.0359 |
| 18:0 | 39.65 | 6.57 | 0.1295 | 6.46 | 0.0536 | 2.79 | 0.0212 | 3.13 | 0.0508 |
| 19:0 | 40.66 | NA | NA | NA | NA | NA | NA | NA | NA |
| 19:0Δ | 41.10 | 6.65 | 0.0333 | 6.89 | 0.0769 | NA | NA | NA | NA |
| 20:0 | 43.96 | NA | NA | NA | NA | NA | NA | NA | NA |
| 22:5 | 45.09 | NA | NA | NA | NA | NA | NA | NA | NA |


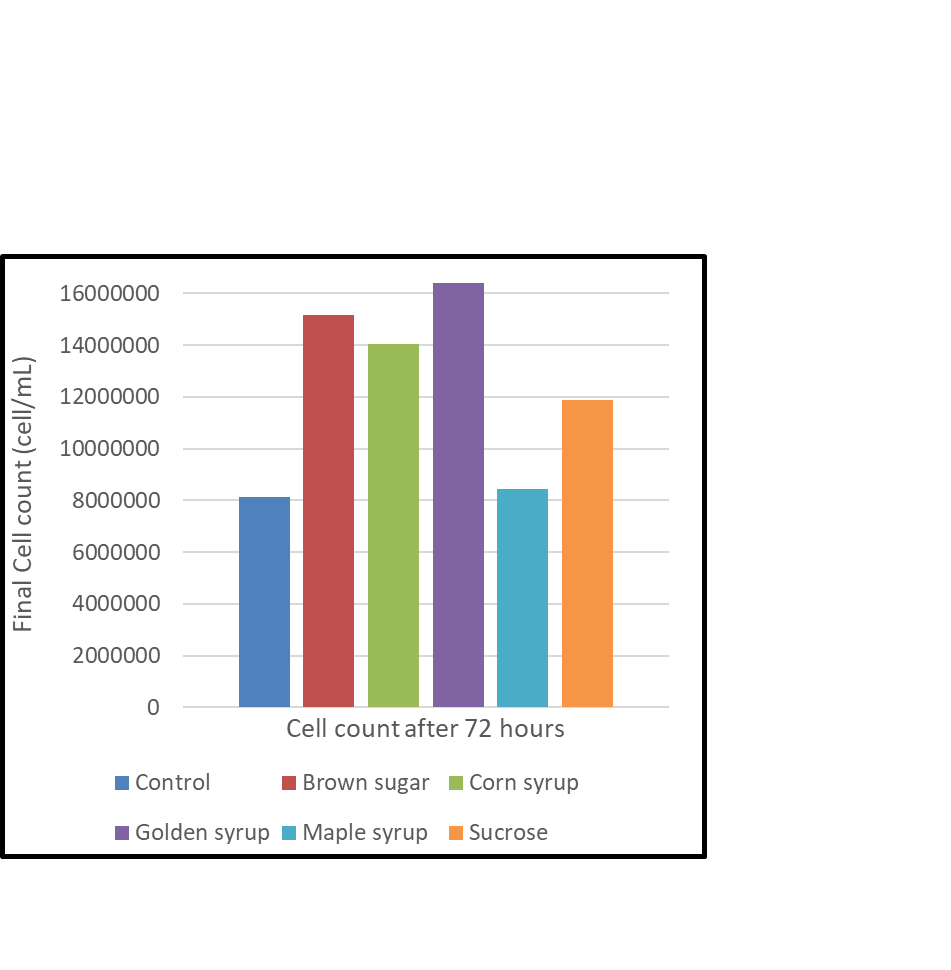


Figure S6. Cell density of *C. fasciculata* WT cultured upon supplementation with commercial sugars. The bar chart shows the final cell density (Y axes) at the end of the growth curve performed for *C. fasciculata* WT cultured in media supplemented with various sugar sources (X axis) at 27˚C for 72 h.

Table S14. Fatty acid content in *C. fasciculata* WT cultured in media supplemented with brown sugar and golden syrup at 27°C. The table shows a summary of the relative abundance and the retention times of the FAMEs or FAs after GC-MS analysis of samples obtained from *C. fasciculata* WT control grown in media supplemented with brown sugar or golden syrup or sucrose at 27°C for 48 h. Values are the mean of three independent biological replicates (n=3). SD is standard deviation of each mean (±).

|  |  |  |  |  |  |  |  |  |  |
| --- | --- | --- | --- | --- | --- | --- | --- | --- | --- |
|  |  | WT Control 27°C | | WT Brown Sugar 27°C | | WT golden syrup 27°C | | WT Sucrose 27°C | |
| FAME | Time (min) | Relative Abundance (%) | SD | Relative Abundance (%) | SD | Relative Abundance (%) | SD | Relative Abundance (%) | SD |
| 12:0 | 31.67 | 8.22 | 0.1316 | 5.58 | 0.2147 | 4.52 | 0.1652 | 7.15 | 0.0903 |
| 14:0 | 33.02 | 8.41 | 0.1027 | 6.79 | 0.2013 | 7.69 | 0.2275 | 9.46 | 0.0160 |
| 15:0 | 34.91 | 2.36 | 0.1446 | 3.95 | 0.2379 | 2.35 | 0.1620 | 2.82 | 0.0522 |
| 16:2 | 35.37 | NA | NA | 3.16 | 0.1314 | NA | NA | NA | NA |
| 16:1 | 35.81 | 1.34 | 0.1051 | 1.59 | 0.0967 | NA | NA | NA | NA |
| 16:0 | 37.26 | 7.69 | 0.1126 | 6.72 | 0.2080 | 6.71 | 0.2837 | 9.11 | 0.0257 |
| 17:0 | 37.72 | 2.43 | 0.1065 | 3.81 | 0.1444 | NA | NA | NA | NA |
| 17:0Δ | 38.81 | NA | NA | NA | NA | NA | NA | 1.84 | 0.0672 |
| 18:3 | 38.86 | 4.45 | 0.2653 | 5.82 | 0.1447 | 4.95 | 0.0818 | 2.50 | 0.0159 |
| 18:2 | 39.34 | NA | NA | 2.58 | 0.2030 | 6.83 | 0.1495 | 5.43 | 0.2780 |
| 18:1 | 39.35 | NA | NA | 34.55 | 0.2258 | 46.17 | 0.4227 | 50.27 | 0.7104 |
| 18:1 | 39.65 | NA | NA | NA | NA | NA | NA | NA | NA |
| 18:0 | 40.66 | 5.66 | 0.0664 | 6.83 | 0.0419 | 6.54 | 0.2108 | 4.14 | 0.0158 |
| 19:0 | 41.10 | NA | NA | NA | NA | NA | NA | NA | NA |
| 19:0Δ | 43.96 | 6.31 | 0.1796 | 7.70 | 0.1335 | 6.57 | 0.0308 | 4.86 | 0.0483 |

**A**

**B**

**C**

**D**

**E**

Figure S7. Comparison of fatty acids produced by *C. fasciculata* after supplementation with commercial sugars.  The bar chart aims to give a visual summary of the of variation of FAs (Y axis, the order follows increasing retention time) comparing the total of each class of FAs (X axis, part of total obtained by contingency table produced with PRISM 6.0) in C. fasciculata cultured in minimal media without Tween-80 and sugar, and *C. fasciculata* grown in minimal media supplemented with 750 μg of corn syrup, maple syrup, golden syrup, brown sugar and sucrose.
